# Impact of fluoxetine exposure on *Lymnaea stagnalis* and its developing eggs: integrating untargeted lipidomics, targeted metabolomics, and classical risk assessment

**DOI:** 10.3389/fphar.2025.1536438

**Published:** 2025-02-04

**Authors:** Diana Ilyaskina, Yumi Nakadera, Marja H. Lamoree, Joris M. Koene, Pim E. G. Leonards

**Affiliations:** ^1^ Amsterdam Institute for Life and Environment (A-LIFE), Vrije Universiteit Amsterdam, Amsterdam, Netherlands; ^2^ Senckenberg Ocean Species Alliance, Senckenberg Research Institute and Natural History Museum Frankfurt, Frankfurt, Germany

**Keywords:** fluoxetine, *Lymnaea stagnalis*, lipid metabolism, ecotoxicology, neurochemical pathways

## Abstract

Pharmaceuticals such as selective serotonin reuptake inhibitors (SSRIs), are increasingly detected in aquatic environments, posing potential risks to non-target organisms, because many of those substances are widely shared neuromodulator. In this study, we investigated the effects of SSRI antidepressant, namely, fluoxetine, exposure on the freshwater snail *L. stagnalis*, focusing on egg development, neurochemical pathways, and lipid metabolism. Snails were exposed to a range of 51–434 µg fluoxetine L⁻^1^ for 7 days, followed by analysis of survival, feeding behaviour, reproduction, and metabolomic changes in the central nervous system (CNS), albumen gland, and eggs. Although no significant effects were observed on survival or fecundity, fluoxetine exposure significantly impaired egg development in a dose-dependent manner, reducing hatching rates with an EC50 of 126 µg fluoxetine L⁻^1^. Removal of eggs from the contaminated environment partially reversed these developmental effects, suggesting potential recovery if fluoxetine levels decrease. Molecular analysis revealed several neurochemical and lipidomic alterations. In the CNS, elevated levels of catecholamines, phosphatidylcholines (PC), and ceramides were linked to disruptions in neurotransmission, membrane integrity, and impaired embryo development. In the albumen gland, we detected a decrease of key lipid classes, including sphingomyelins and fatty acids, which can be linked with impaired egg quality. Additionally, a decrease in histamine in both the albumen gland and eggs suggested further disruption of egg development, potentially affecting metamorphosis success. Moreover, the dose-dependent increase in choline, along with PC and oxidized PC, indicated oxidative stress and lipid peroxidation in the CNS and exposed eggs of *Lymnaea stagnalis*. Our findings highlight the benefits of combining behavioral assessments with metabolomic profiling to better understand the mechanistic pathways underlying fluoxetine’s adverse effects.

## 1 Introduction

Antidepressant pharmaceuticals are among the most consumed and prescribed drugs worldwide (Imiuwa et al., 2024; Lewer et al., 2015; Luo et al., 2020). A prominent class of antidepressants, selective serotonin reuptake inhibitors (SSRIs), notably fluoxetine (Prozac), has gained wide usage due to its capacity to modulate serotonin distribution in the central nervous system (CNS) (Castillo-Zacarías et al., 2021). The main formulations and their metabolites target neurotransmitter pathways and their membrane transporters, which are evolutionary conserved across diverse animals (Imiuwa et al., 2024; Tierney, 2018). As antidepressants are widely detected in water (from ng to low µg active substance L^-1^), this can potentially lead to a chronic exposure in aquatic species and pose a significant risk to aquatic ecosystems (Castillo-Zacarías et al., 2021; Imiuwa et al., 2024; Souza et al., 2022). Although neurotransmitters like serotonin are widespread in the animal kingdom and present in aquatic species, the effects of SSRI exposure in invertebrates can vary widely, both at the molecular level–through alterations in neurotransmitter pathways–and at the phenotypical level, such as changes in behavior, reproduction, and development, which potentially need further assessment (Baynes et al., 2019).

Gastropods are excellent taxa to examine the impact of antidepressants, as they are prevalent in both marine and freshwater ecosystems and play a crucial role in ecosystem balance. In this study, we used the great pond snail, *L. stagnalis*, a freshwater gastropod that has emerged as a model organism for ecotoxicological studies due to its biological functions and documented sensitivity to contaminants (Fodor et al., 2020). Notably, as a simultaneous hermaphrodite that produces transparent eggs, *Lymnaea stagnalis* offers insights into both male and female reproductive systems and allows for the direct observation of egg development and the success rate of metamorphosis under exposure conditions (Fodor et al., 2020). Observations of altered behavioral patterns in aquatic species, including changes in reproduction and feeding, suggest profound underlying molecular disruptions caused by pollutants like fluoxetine (Weinberger and Klaper, 2014). These behavioral changes are important in classical risk assessments, guided by OECD 243 (OECD, 2016). In addition to adult fitness, egg development and hatching success have been used as indicators of ecological factors for population health and growth under stress conditions and chemical exposure (Weinberger and Klaper, 2014).

To gain insight into the biological responses on a molecular level, metabolomics has become a valuable tool, capturing the real-time biochemical profile of an organism, and revealing the metabolic pathways disrupted by exposure to pollutants. Since metabolites are end-products of cellular processes, they serve as a direct indicator of stress-induced effects on organisms (Viant et al., 2019). Assessing of metabolites like lipids, amino acids, and neurotransmitters provides valuable input into toxicological studies, linking molecular alterations with phenotypic adverse outcomes, and providing detailed information for decision making and chemical regulation (Tufi et al., 2016; Xu et al., 2022). For example, long-term (14 weeks) exposure of *L. stagnalis* to environmental concentrations of diclofenac, a nonsteroidal anti-inflammatory drug, has demonstrated significant alterations in adenine, tryptophan, and uridine levels, as well as impaired growth, indicating disruptions in key metabolic pathways with potential ecological consequences (Bouly et al., 2022). Similarly, recent studies have shown the potential effects of fluoxetine and other contaminants on the behavior and physiology of aquatic organisms (Benatti et al., 2017; David et al., 2018; Weinberger and Klaper, 2014).

Among studied metabolites, neurotransmitters and amino acids play particularly pivotal roles in regulating behavior, reproduction, and neural development, as well as mediating responses to environmental stressors (Bacqué-cazenave et al., 2020). Understanding their dynamics offers profound insights into the neurodevelopmental and adaptive mechanisms of aquatic organisms exposed to environmental contaminants. Amino acids and neurotransmitters can influence multiple aspects of adaptive fitness in the adult animals as well as the development of their offspring. Several studies applied molecular tools to address biological functions of metabolites in *L. stagnalis*. A comprehensive study by Ivashkin et al. (2015) showed the importance of serotonin in *L. stagnalis* impacting early pre-neural developmental stages, influencing embryonic and juvenile growth, feeding behavior, and locomotion dynamics within the veliger stage, and during the entire development of *L. stagnalis* up to 25 days after hatching (Ivashkin et al., 2015). Notably, previous metabolomics studies have shed light on pesticide-induced toxicity in the CNS of *L. stagnalis*, as seen in the study exploring the effects of imidacloprid (Tufi et al., 2015). Moreover, recent studies have identified serotonin transporters (SERTs), responsible for the reuptake of serotonin in *L. stagnalis* and demonstrated its localization within serotonergic neurons, such as cerebral giant cells and pedal ganglia neurons (Chikamoto et al., 2023). SERT expression levels in the CNS correlated with nutritional states, with lower expression under severe food deprivation (Chikamoto et al., 2023). Given that fluoxetine is a SSRI, targeting SERT activity, it is likely to interfere with serotogenic pathway in the snails as well.

The current study aims to investigate the impact of fluoxetine exposure on adult *L. stagnalis* and their egg development, using targeted and non-targeted metabolomics approaches and classical assessments of reproduction, feeding, egg development and hatching success. This study also evaluates the recovery potential of *L. stagnalis* embryos, produced by exposed adults and initially exposed for 2 days, by transferring them to uncontaminated water for further development.

## 2 Materials and methods

### 2.1 Chemicals and reagents

Acetonitrile (ACN), 2-propanol (IPA), chloroform, and methanol (MeOH) were sourced from Biosolve B.V., Valkenswaard, Netherlands. Ethanol (EtOH) of analytical grade was acquired from Sigma-Aldrich, Germany. Milli-Q water was produced using a Millipore purification system (Merck KGaA, Darmstadt, Germany), specifically through the Q-POD Ultrapure Water Remote Dispenser. The isotope-labelled internal standard (IS) SPLASH LIPIDOMIX Mass Spec Standard was procured from Avanti Polar Lipids, Alabaster, AL, United States. Both Fluoxetine (CAS 56296-78-7) and the IS Fluoxetine-d6 (free base concentration of 100 μg IS mL^-1^ in methanol), were purchased from Sigma-Aldrich, Germany. The full list of measured neurotransmitters and its isotope-labeled ISs are shown in the Supplementary material (Supplementary Table S1).

### 2.2 Test animals

The laboratory strain of *L. stagnalis*, maintained at Vrije Universiteit Amsterdam, was utilized to examine the impact of fluoxetine on life history traits and metabolomic responses. The snails were originally collected nearby Utrecht, the Netherlands, ca. 60 years ago. The snail cultures are maintained in large flow-through tanks with low concentrations of copper in the water, at a controlled temperature of 20°C ± 1°C and a light-dark cycle (L:D) of 12:12 h. Their diet consists of broad leaf lettuce and fish food (TetraPhyl, Tetra GmbH). Adult *L. stagnalis* lay 2-3 egg masses per week, with each egg mass comprising 80-120 eggs. These snails can store sperm from mating partners for about 3 months (Cain, 1956; Nakadera, 2014).

To examine the effects of fluoxetine on egg development, we collected age-synchronized egg masses by placing glass bottles in a flow-through tank with a mass culture for 4–5 h. Because the snails prefer to lay eggs on a clean surface (referred to as the Clean Water Stimulus, (Ter Maat et al., 1989)), the snails are immediately triggered to lay eggs, and the bottle surface becomes covered with freshly laid egg masses after several hours. Since the same method is used for mass culture maintenance, the adult snails are also age-synchronized.

### 2.3 Experimental design

#### 2.3.1 Adult test

**FIGURE 1:**
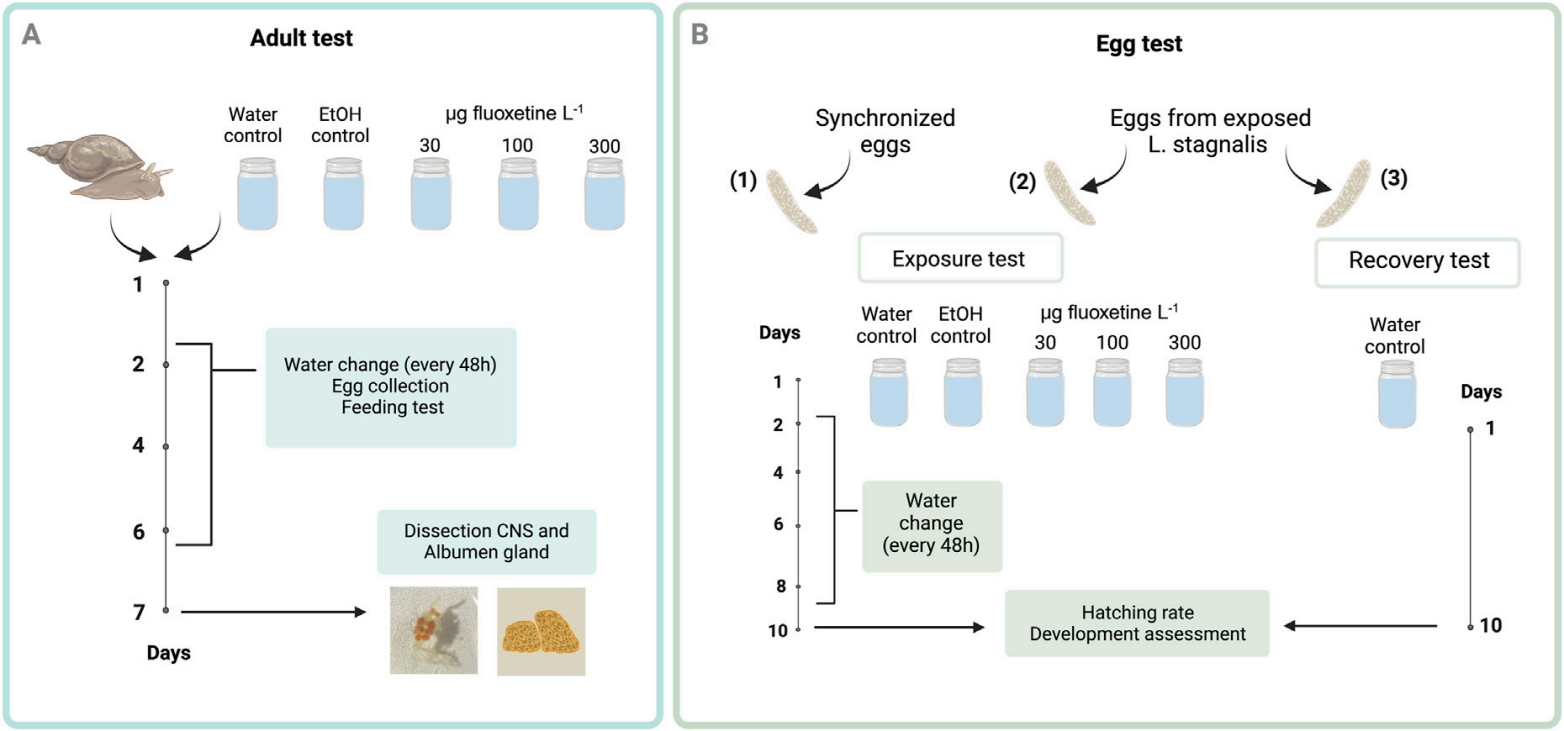
Schematic representation of the experimental design to evaluate fluoxetine effects on *Lymnaea stagnalis* **(A)** and their eggs **(B)**. In Panel A, adult snails were exposed to three fluoxetine concentrations with water changes every 48 h, egg collection for exposure and recovery tests, and feeding tests. After 7 days, the CNS and albumen gland were dissected for analysis. In Panel B, eggs were similarly exposed, with water changes every 48 h. After 10 days, hatching rates and developmental progress were assessed to determine fluoxetine’s impact on early life stages. The exposure concentrations presented in the experimental design are nominal values. Created in https://BioRender.com.

Acclimatization and experimental procedures for snails were conducted in accordance with OECD 243 guidelines, with modifications, using low-copper water (OECD, 2016). Age-synchronized adult *L. stagnalis* (3 months old, N = 75) were used in the exposure to fluoxetine for 7 days. Prior to exposure, each snail was placed individually in a glass jar containing approximately 300 mL of low-copper water, undergoing a 3-days acclimatization period within a climate-controlled environment (20°C, L:D = 12:12 h). Three nominal concentrations of fluoxetine were used: 30, 100, and 300 µg Fluoxetine L^-1^. Initially, fluoxetine spike solution was prepared in ethanol, then added to fresh low-copper water to achieve the desired concentrations. Control groups included water only (C) and another with water spiked with an ethanol volume equivalent to that in the High fluoxetine concentration (E). Each treatment had 15 replicates. The snails were transferred into new glass jars with freshly spiked water every 2 days. The detailed exposure protocol for *L. stagnalis* is illustrated in Figure 1A. The feeding, egg collection, and water change procedures are described in detail below.

#### 2.3.2 Egg test

The effects of fluoxetine exposure on eggs were examined using three types of eggs: (1) synchronized eggs from non-exposed snails, (2) eggs laid by the exposed snails and incubated in the same spiked water as their mother, and (3) eggs laid by exposed snails and incubated in clean water (recovery) (Figure 1B). The rationale of examining the eggs from non-exposed snails is to test if fluoxetine affects the development of snails, and if so, to obtain the dose response curve. Age-synchronized egg masses (N = 50, with 10 replicates for each treatment) were collected and each egg mass was placed in a small glass jar containing either clean or spiked water (15 mL). To monitor all developmental stages of the embryos during development and metamorphosis, eggs were exposed for 10 days, aligning with their approximate 14-day developmental period at 20°C. The egg test was conducted under the same climate-controlled environment used for the adult test, with water exchanges occurring every 2 days. Additionally, egg masses from exposed adult snails were collected to assess the potential to recover from fluoxetine exposure. Egg masses laid by the exposed snails on day 3, 5, and 7 were harvested. Each egg mass was bisected using forceps, with one-half incubated in spiked water as per the maternal treatment and the other in clean, low-copper water. A previous study showed that breaking the egg mass wall does not hinder egg development of this species (Marois and Croll, 1991). Then, all the egg masses were incubated in the small glass jars for 10 days. The protocol for the *L. stagnalis* eggs exposure test is described in Figure 1B.

#### 2.3.3 Sample collection

At the end of the experiment, adult snails were dissected to extract the albumen gland and CNS for metabolomic analysis. Approximately 2 mL of 50 mM MgCl_2_ was injected into the foot using a thin needle and syringe to anesthetize the snails. After removing the shell using forceps, the skin above the female gonopore was incised to access the albumen gland (3–5 mm in diameter), which was then placed into a 1.5 mL Eppendorf tube. The incision was extended towards the head to access the CNS, and all connecting nerves were severed with dissection scissors before placing the CNS (2–3 mm in diameter) into a tube. Upon concluding the exposure of eggs laid by non-exposed snails, 10 individual eggs were taken from egg masses of each replicate for metabolomic analysis. All samples were snap-frozen in liquid nitrogen and stored at −80°C prior to extraction.

### 2.4 Toxicity endpoints

#### 2.4.1 Feeding, reproduction and egg development

To assess the impact of fluoxetine on adult snails, their feeding behavior and fecundity were measured. After the water change for exposed and control animals, a lettuce disc (area: 19.6 cm^2^) was given to each snail. Lettuce leaves were shaped into discs using a metal ring, and the provided food amount was slightly less than the daily food intake of an adult snail (Zonneveld and Kooijman, 1989). Approximately 20 h later, the leftover lettuce was collected and digitized using a flatbed scanner (Canon LiDE 220, 1,200 dpi). Subsequently, the snails were offered another lettuce disc for that day. The area of the remaining lettuce was quantified using ImageJ (ver. 1.53 k) (Koene et al., 2010). To determine the fecundity of snails exposed to fluoxetine, egg masses produced during the exposure period were collected and imaged using the same scanner (van Iersel et al., 2014). The number of eggs was counted utilizing the scanned images with ImageJ.

The influence of fluoxetine on egg development was examined by evaluating the hatching rate and developmental stages. Egg masses, after 10 days of exposure or recovery, were visualized using the flatbed scanner. Developmental stages were categorized into three groups: Developed eggs (corresponding to 10 days of embryogenesis), Postmetamorphic stage (equivalent to 7 days of embryogenesis), and Non-developed (Veliger stage, equating to three to 4 days of development, along with non-developed embryos) (Ivashkin et al., 2015). To provide clarity on the methodology, example photographs of scanned egg masses used for developmental stage classification and hatching rate analysis in ImageJ software are included in the Supplementary Material (Supplementary Figure S1).

#### 2.4.2 Lipidomics, neurotransmitters and amino acids analysis: sample preparation

To perform extraction of lipids, neurotransmitters, and amino acids solid-solvent extraction was conducted according to Xu et al. (2022). Briefly, 150 µL of ice-cold milli-Q water was added to each sample, followed by two 10-s cycles at 6,500 rpm using a Precellys 24 Dual device (Bertin Technologies, France). Each sample consisted of one CNS, one albumen gland, or 10 pooled eggs per replicate, as described in the Sample Collection section. Next, 10 µL of each homogenate was taken and placed in a 96-well plate for protein quantification using the Bradford assay, to normalize metabolite concentrations across samples. Further, 150 µL of ACN containing neurotransmitters mix IS was added to the remaining homogenates, and the mixture was homogenized again using the Precellys. After adding 290 µL of chloroform and 10 µL of lipid IS, Precellys cycles were repeated. The samples were left for 10 min on ice for protein precipitation followed by centrifugation for 8 min at 14,000 rpm. The extracts formed two distinct layers: a polar metabolite-rich upper layer and a non-polar lipid-rich bottom layer.

To analyze the neurotransmitters and amino acids, 150 µL of the upper layer was transferred to a 1.5 mL Eppendorf tube and centrifuged for 5 min at 14,000 rpm. Then samples were evaporated to dryness using a CentriVap vacuum concentrator (Labconco) at 15°C for 2 h. Finally, samples were reconstituted in 100 µL of ACN:milli-Q water (90:10, v/v). To analyze lipids, 200 µL of the bottom layer was transferred to a glass vial and evaporated under a light nitrogen flow till dryness. Then, samples were reconstituted with 100 µL ACN:milli-Q water:IPA (5:4:2, v/v). All samples were stored at −80°C before the analysis.

#### 2.4.3 Lipidomics, neurotransmitters and amino acids analysis

For lipid analysis, a combination of High-Performance Liquid Chromatography (HPLC) Agilent 1,290 Infinity (HPLC system, United States), and quadrupole Time-of-Flight mass spectrometer (QTOF, Bruker United States) were used. The conditions for separation and detection were optimized according to Xu et al. (2022). Lipids were detected with electrospray ionization in positive and negative modes (ESI+, ESI-). A Kinetex EVO C18 column (100 × 2.1 mm, 2.6 µm particle size, Phenomenex, United States) column was applied for HPLC, at 40°C. Separation of lipids was achieved using the following mobile phases: 1) ACN:milli-Q water (60:40), 10 mM NH4 formate and 0.1% formic acid, and 2) IPA:ACN (90:10), 10 mM NH4 formate with 0.1% formic acid. For the QTOF, data-dependent acquisition was conducted with a scan range of m/z 60-2000, a scan speed of 6 Hz, and a resolution of 25,000 FWHM (full width at half maximum). Before sample injection, mass calibration was accomplished by injecting a sodium formate solution.

To detect neurotransmitters and amino acids, a combination of HPLC (Bruker), and tandem Mass Spectrometry (MS/MS, EvoQ, Bruker) was used. The neurotransmitters and amino acids were detected with ESI in positive mode. The MS/MS settings were as follows: spray voltage 3500 V, cone temperature 350°C, heated probe temperature 300°C, nebulizer gas flow 60 units. Separation was achieved using an XBridge Amide column (150 × 2.1 mm, 3.5 µm particle size, Waters, United States) at 40°C. Mobile phases consisted of ACN:milli-Q water (5:95, v/v) with 100 mM ammonium formate (A) and ACN:milli-Q water (85:15, v/v) with 30 mM ammonium formate (B), both adjusted to pH 3. The gradient for mobile phases was the following: gradient of A from 5% to 45% in 9 min, followed by 5 min of washing with 50% of A, and further column equilibration for 15 min with 5% of A. The flow rate was 0.4 mL min^-1^, and the injection volume was 10 µL. The calibration curve was built with seven levels and started from 0.4 to 1,600 ng mL^-1^. Linearity of calibration curve, limit of detection (LOD) and quantification (LOQ) of measured compounds, that reflect signal to noise ratio (S/N) of 3 and 10, subsequently, are presented in the Supplementary material (Supplementary Table S2). A detailed description of multiple reaction monitoring (MRM) transitions for neurotransmitters and their IS are presented in the Supplementary material (Supplementary Table S1).

### 2.5 Fluoxetine quantification

To quantify fluoxetine in water at the beginning and after 48 h, 1 mL of water was taken at each round of water change. Further, the water samples were centrifuged at 14,000 rpm at room temperature for 5 min and 500 mL of supernatant was taken for further analysis. Next, 50 µL of the IS fluoxetine-d6 was added to each sample. Quantitative analysis was performed using an Acquity UPLC system coupled to a Xevo TQ Absolute MS instrument (Waters Corporation, United States). The MS system was operated using ESI, both positive and negative modes. The Omega Luna 1.6 μm PS C18 (50 × 2.1 mm) column was used for the separation. The mobile phases were milli-Q water (A) and methanol (B) both with 0.05% formic acid. The gradient for fluoxetine was as follows: 0.2 min of 100% of A, followed by 4 min decrease of A till 21%, with a further wash step for 7 min (0% A), and 3.5 min of stabilizing the column with 100% of A. The column temperature was 40°C and the flow rate 0.5 mL min^-1^. The injection volume was 5 µL. The retention time for fluoxetine and fluoxetine-d6 was 2.9 min. Mass transitions were 310.11 → 44.01 and 148.07 for fluoxetine, and 316.11 → 44.01, 95.16, and 154.1 for fluoxetine-d6. MS system operated with following settings: capillary voltage 2.50 kV, cone voltage 30 V, collision energy 8, source temperature 150°C, desolvation temperature 50, desolvation gas flow 300 L h^-1^, nebulizer 7.0 Bar. LOD and LOQ were 0.025 and 0.083 ng mL^-1^, subsequently. The calibration curve was prepared with 11 points with a fluoxetine concentration range of 0.01–18.79 ng mL^-1^. The linearity was estimated as *R* ^*2*^ = 0.993. The recovery of the extraction procedure was 102.2% ± 9.3% (standard deviation, n = 8) and 88.7% ± 9.2% (standard deviation, n = 8) for fluoxetine-d6 and fluoxetine, respectively. Acquired data of the calibration curve and experimental samples were processed with TargetLynx software (v. 4.1.1.0). The concentrations represented in plots and further analysis were calculated as an average between the initial concentration right after spiking and the fluoxetine concentration measured after 48h, since the average recovery of fluoxetine after 2 days was 77.8% ± 13.4% (standard deviation, n = 14). The quantification data are shown in the Supplementary material (Supplementary Table S3). For the CNS and albumen gland of *L. stagnalis*, actual exposure concentrations of fluoxetine in water were Low - 51, Medium - 135, and High - 434 µg fluoxetine L^-1^. For eggs, actual exposure concentrations of fluoxetine in water were Low - 53, Medium - 137, and High - 417 µg fluoxetine L^-1^.

### 2.6 Quality control, data processing and statistical analysis

#### 2.6.1 Feeding, reproduction, and egg development

The feeding behavior was analyzed using a linear mixed-effects model (LMM) with Treatment (Control, EtOH, three concentrations [Low, Medium, High]) and Day (Day 2, 4, 6) as fixed factor and sample ID as random factor. The total number of eggs that adult snails laid was fitted to the LMM with two fixed factors (Treatment, Day) to run an ANOVA.

For the egg development, we calculated the hatching rate by using all the eggs in an egg mass, and the number of developed eggs. These developed eggs were not exactly hatched at that moment, but due to their advanced developmental stages compared to postmetamorphic or non-developed ones, we assumed that they would have hatched if we let them develop a few more days. For the measurements of the hatching rate, we fitted the data using a generalized linear model (GLM) with binomial distribution and set Treatment as fixed factor. We consider a binomial distribution appropriate as the total number of eggs and the number of developed eggs are count data. When needed, we further conducted *post hoc* Tukey tests. For the hatching data of eggs laid by non-exposed snails, we combined the data of water and EtOH control groups as the group of zero fluoxetine. Moreover, since there was no variation in this dataset, we removed the data of the High concentration group to run a *post hoc* test.

Log-logistic concentration–response curves were built with data observed on exposed eggs of *L. stagnalis* to fluoxetine using the R package drc version 3.0.1 (Ritz and Streibig, 2005). The model was fitted to represent the dose-response relationship between measured concentrations of fluoxetine and hatching rate of eggs. The models were used to estimate the EC50 value and corresponding 95% confidence intervals (CI).

To test if the effects of fluoxetine on development are recoverable, we have two datasets - the data from eggs incubated in spiked water and the data incubated in water (Figure 1B). Again, we used GLMs with binomial distributions, and the models contained Treatment and Day as fixed factors. When needed, we further conducted *post hoc* Tukey tests. Additionally, combined GLM analysis of the two datasets from exposed and recovery groups of eggs was considered to assess the overall impact of fluoxetine treatment. This analysis was not the primary focus of the study due to inherent differences in the biological scenarios, which are better represented through separate analyses. Nonetheless, a combined GLM model has been provided as an alternative analysis and the corresponding R script is available on Zenodo repository (https://doi.org/10.5281/zenodo.14235510). All the statistics were conducted in R (ver 4.2.2, R Core Team 2024).

#### 2.6.2 Lipidomics

To ensure the maintenance of laboratory standards, quality control procedures have been implemented to guarantee the accuracy, precision, and validity of the results. Primarily, the background noise and clarity of baseline, IS recoveries, and stability of retention time were assessed using procedural blanks (PB) and neat blanks (NB). For NB, the lipid IS mixture was added to ACN and directly measured. For PB, lipid IS were added to 150 mL of water and further extracted using the same procedure as the samples, then evaporated to dryness, reconstituted in a mixture in ACN:milli-Q water:IPA (5:4:2 v/v), and subsequently analyzed. Further, quality control (QC) samples were prepared for each analyzed snail organ and eggs, combining 10 μL of each replicate. The QC samples were injected at the beginning of the sample batch, after every ninth sample, and at the end of the batch to monitor any potential shift in retention time and fluctuations in the intensity of mass spectrometer drifts throughout the sequence. The QC samples were used to normalize data within the batch run. During the analysis, a manual assessment of the data was conducted to ensure accurate peak detection and integration.

The chromatograms obtained from lipidomics analysis were calibrated by mass with Bruker Data analysis and converted into MZML format. To identify lipid features, MS-DIAL was used. MS-DIAL is open-source software that can match isotope patterns, exact mass, and MS/MS fragments by using the LipidBlast mass library (Tsugawa et al., 2015). A full description of the settings used in the software is presented in Supplementary Table S4. Lipidomics, neurotransmitters, and amino acids data were normalized using protein content measured in every replicate. The protein content measurements can also be found in the Supplementary material (Supplementary Table S4). The graphs and tables were made with Metaboanalyst 5.0 and in R (ver 4.2.2, R Core Team 2024) to present the obtained data understandably. Metaboanalyst 5.0 was used principal component analysis (PCA) to visualize overall clustering patterns, and partial least squares-discriminant analysis (PLS-DA) to identify variable importance in projection (VIP) features with VIP >1 (Pang et al., 2022). Additional analyses included Two-way ANOVA (FDR corrected), Volcano plots (univariate analysis), fold change analysis, and heatmaps (based on Ward clustering analysis). Fold change was calculated by dividing the levels of metabolites in the exposure groups by those in the control group.

#### 2.6.3 Neurotransmitters and amino acids

To ensure the quality performance, several blank samples (methanol) were used in the sequence of measurements to check for carry over from the experiment measurements. No trace of neurotransmitters was detected in the blank chromatograms. Acquired data of the calibration curve and experimental samples were processed with MS Data Reviewer (Bruker). Measured concentrations of neurotransmitters and amino acids were further normalized by protein content of each replicate for each *L. stagnalis* organ and its eggs. The two-way ANOVA, with further Tukey *post hoc* analysis, was performed with a *p*-value <0.05. and boxplots were generated in R (ver 4.3.1).

## 3 Results

### 3.1 Feeding and reproduction

All the adult snails survived the exposure of 7 days. Five individuals did not lay any eggs during the exposure, so we removed them from further analyses (two water controls, two EtOH, one Medium exposure replicate). For the feeding behavior, there was no effect of treatments (LMM, *F*_4, 65_ = 1.01, *p* = 0.411), although the snails altered feeding behavior over time (LMM, *F*_2, 129_ = 42.56, *p* < 0.001, Supplementary Figure S2) but this was irrespective of treatment. Fecundity, in terms of total number of eggs of *L. stagnalis* was also not affected by treatment (ANOVA, *F*_4, 220_ = 0.21, *p* = 0.932) nor sampling day (*F*_2, 218_ = 0.03, *p* = 0.969: Supplementary Figure S3).

### 3.2 Eggs development

**FIGURE 2:**
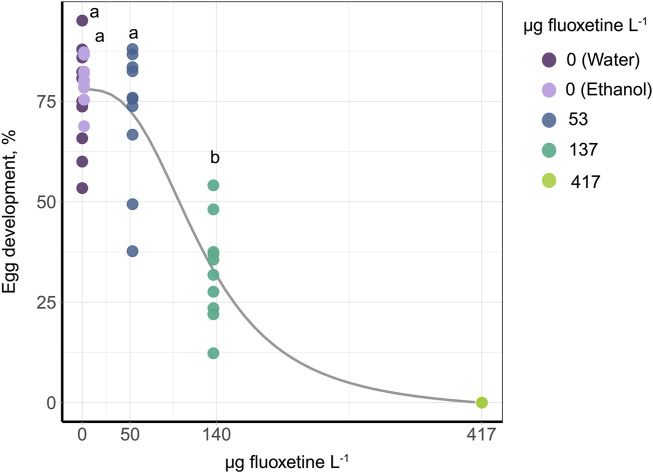
Dose response curve of egg development after 10 days of fluoxetine exposure. The control group of water and EtOH is combined as zero concentration. In order to run a *post hoc* test, the data of 417 µg fluoxetine L^− 1^ were removed as there was no variation. The outcome of the *post hoc* test is shown as the letters above the data points, with different letters indicating significance between concentrations.

Egg development was significantly affected by the fluoxetine exposure. The 10 days exposure of the synchronized eggs showed significantly reduced hatching rate (GLM, Deviance = 763.36, *p* < 0.001) and EC_50_ was 126 µg fluoxetine L^-1^ (95% CI: 106–146 µg fluoxetine L^-1^). The slope of the dose-response curve, as derived from the Hill function, was 2.88 (Figure 2).

**FIGURE 3:**
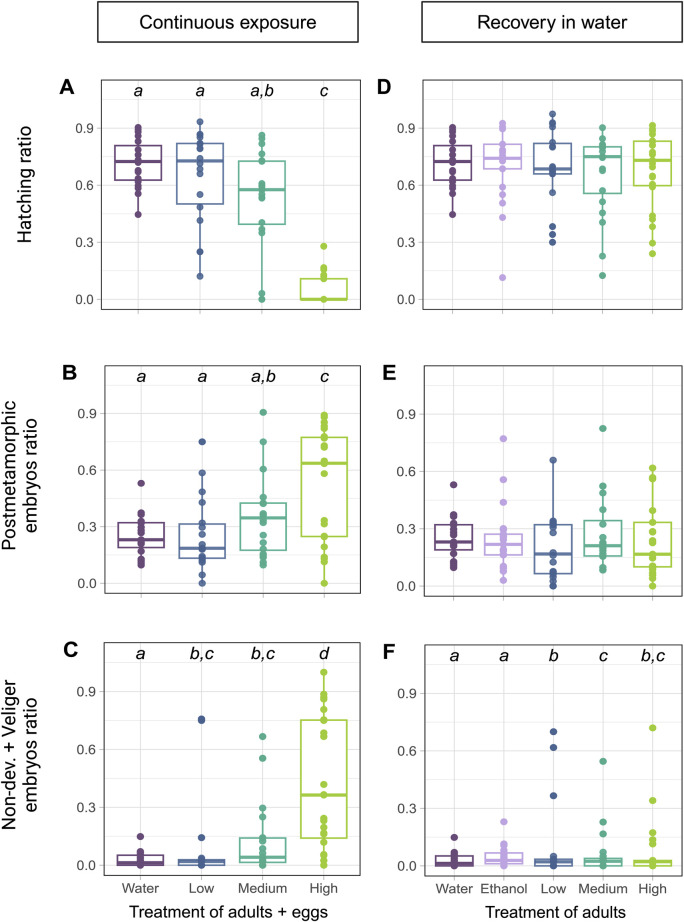
The effects of fluoxetine on egg development and their recovery. **(A–C)** Eggs laid by exposed snails and incubated in spiked water. The control groups (water and ethanol) were pooled in these analyses to simplify comparisons, as no significant differences were observed between the two controls (*p* > 0.05). Concentration levels are Low – 53, Medium – 137, and High – 417 µg fluoxetine L^-1^. **(D–F)** Eggs laid by exposed snails and kept incubated in water. **(A, D)** Hatching success expressed as ratio of the total number of eggs in the egg mass. **(B, E)** The ratio of postmetamorphic embryos. **(C, F)** The ratio of non-developed eggs and veliger embryos. The outcome of the *post hoc* test is shown as the letters above the data points, with different letters indicating significance between concentrations.

The eggs laid by exposed snails and kept in spiked water for the next 10 days showed significantly reduced hatching rate in the groups at 137 and 417 µg fluoxetine L^-1^ concentrations (GLM, Treatment: Deviance = 571.54, *p* < 0.001, Day: Deviance = 2.19, *p* = 0.335, Figure 3A), consistent with the data in Figure 2. In addition, the ratio of postmetamorphic embryos differed across treatments (GLM: Treatment: Deviance = 104.21, *p* < 0.001, Day: Deviance = 1.61, *p* = 0.448, Figure 3B), as well as the ratio of non-developed embryos (GLM, Treatment: Deviance = 458.32, *p* < 0.001, Day: Deviance = 4.57, *p* = 0.102, Figure 3C). These outcomes showed that the eggs from Medium and High concentration treatments (137 and 417 µg fluoxetine L^-1^) did not develop properly. In contrast, when the eggs were laid by exposed snails and transferred into clean water to develop for the next 10 days, we did not detect any differences in hatching rate across treatments (GLM, Treatment: Deviance = 1.16, *p* = 0.885, Day: Deviance = 9.58, *p* = 0.008, Figure 3D). Also, the rate of postmetamorphic embryos did not differ significantly in that case (GLM, Treatment: Deviance = 5.35, *p* = 0.253, Day: Deviance = 31.78, *p* < 0.001, Figure 3E). We did observe a significant difference in the ratio of non-developed eggs between treatments, with an increase in the amount of non-developed embryos and embryos reaching only the veliger stage in eggs of exposed snails (GLM, Treatment: Deviance = 33.02, *p* < 0.001, Day: Deviance = 1.56, *p* = 0.459, Figure 3F). These results indicate that the effects of fluoxetine on egg development are partly reversible, with a much lower amount of non-developed eggs in the recovery experiment (Figures 3C, F).

### 3.3 Metabolomic changes in the neurotransmitter and amino acid profiles of *Lymnaea stagnalis* organs and eggs after exposure to fluoxetine

#### 3.3.1 Central nervous system and albumen gland

**FIGURE 4:**
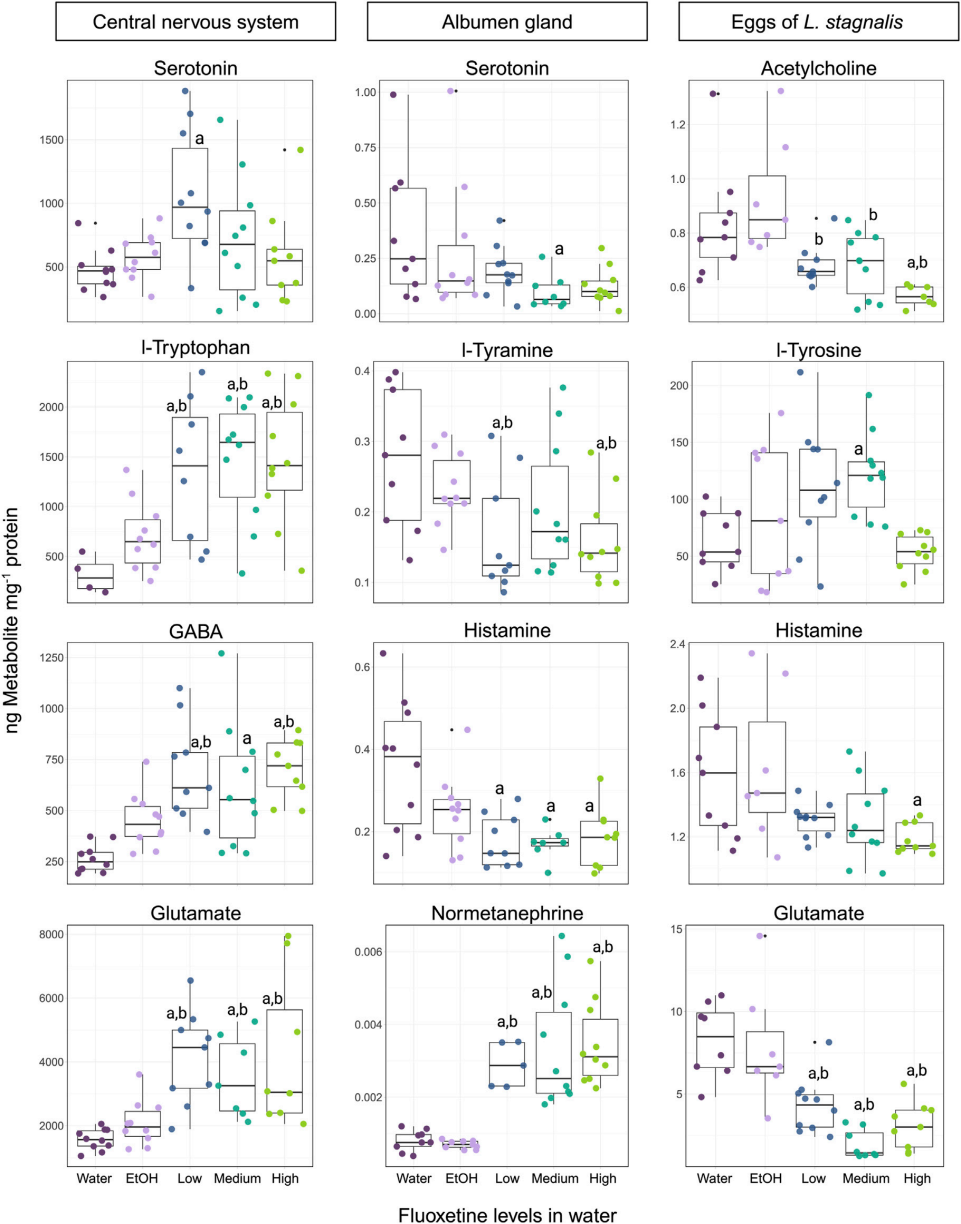
Representation of the four most affected neurotransmitters and animo acids in the CNS, albumen gland, and eggs of *Lymnaea stagnalis* exposed to fluoxetine. For the CNS and albumen gland, concentration levels are Low - 51, Medium - 135, and High - 434 µg fluoxetine L^-1^. For eggs, the exposure levels are 53, 137, and 417 µg fluoxetine L^-1^, respectively. Significant differences (*p* < 0.05) between treatments are indicated by differing letters, where “a” is significance against the water control and “b” – significance against the ethanol (EtOH) control.

The effect of fluoxetine on *L. stagnalis* showed a series of changes that appear in both the CNS and albumen gland. The neurotransmitter and amino acid profiles (12 out of 13 detected and measured) in the CNS were significantly affected at some concentrations of fluoxetine (51, 135, 434 µg fluoxetine L^-1^) (Figure 4; Supplementary Figure S6). Levels of phenylalanine, a precursor to l-tyrosine and, subsequently, to a number of catecholamine neurotransmitters, was significantly increased at all concentrations of fluoxetine compared to the water control group. Further, l-tyrosine levels showed a similar pattern of increase at all fluoxetine exposure groups. Dopamine and epinephrine levels showed concentration-depended increases (Supplementary Figure S6). Levels of L-tryptophan, the precursor to serotonin, exhibited significant increases at all exposure concentration (*p* < 0.05 at 51, 135, 434 µg fluoxetine L^-1^), while the levels of serotonin, a key neurotransmitter involved in a wide range of functions was significantly higher in snails exposed to the Low concentration (51 µg fluoxetine L^-1^), whereas the Medium and High fluoxetine concentrations did not show significant difference to control groups (Figure 4). All amino acids involved in the glutamate/GABA-glutamine cycle were significantly increased in the CNS of exposed *L. stagnalis* (*p* < 0.05 at 51, 135, 434 µg fluoxetine L^-1^; Figure 4). Lastly, levels of choline, histidine and histamine were significantly elevated at all fluoxetine concentrations (Supplementary Figure S6). The fluoxetine-induced modulation of neurotransmitter and amino acid levels in the CNS of snails suggests that the chemical impact extends beyond serotonin to influence a network of neurochemical pathways.

Out of 17 measured neurotransmitters and amino acids in the albumen gland of *L. stagnalis,* 6 were significantly affected (Figure 4; Supplementary Figure S7). Interestingly, the majority of neurotransmitters and amino acids showed decreased levels in the fluoxetine exposure groups. Phenylalanine levels indicated a significant decrease at the 51 µg fluoxetine L^-1^ concentration, as well as for l-tyramine (product of phenylalanine) which showed significant decreases at 51 and 434 µg fluoxetine L^-1^ levels (Figure 4). A significant decrease at the High exposure concentration was observed for dopamine levels, whereas the breakdown product of dopamine, 3-MT (3-methoxytyramine) levels, showed an upward trend with a significant increase at 135 µg fluoxetine L^-1^ concentration compared to the EtOH control group (Supplementary Figure S7). Serotonin levels were significantly lower at the Medium concentration compared to the water control. A downward trend in levels was also observed for histamine at all fluoxetine exposure groups (Figure 4). Normetanephrine, a metabolite of norepinephrine, displayed significant increased levels across all fluoxetine concentrations in comparison to control groups, suggesting that the adrenergic system may be modulated by fluoxetine exposure (Figure 4).

#### 3.3.2 Eggs

Exposure of eggs of *L. stagnalis* to fluoxetine during their development for 10 days resulted in alteration of 6 neurotransmitters and amino acids (out of 11 detected and measured) (Figure 4; Supplementary Figure S8). The cholinergic system was affected by fluoxetine, where levels of acetylcholine exhibited a significant decrease at all fluoxetine concentrations compared to the water and EtOH control groups, while choline levels were notably elevated (*p* < 0.05, 137 µg fluoxetine L^-1^; Figure 4; Supplementary Figure S8). The decrease in histamine (*p* < 0.05, 417 µg fluoxetine L^-1^) and glutamate (*p* < 0.05, all exposure concentrations) levels was observed for eggs of *L. stagnalis* (Figure 4). Levels of l-tyrosine, the amino acid precursor to several catecholamines, were significantly higher in the Medium exposure group (Figure 4). Epinephrine levels demonstrated a notable increase at both 53 and 137 µg fluoxetine L^-1^ concentrations (Supplementary Figure S8). These changes highlight the sensitivity of developing embryos to fluoxetine exposure and underline the need for further investigation into the long-term consequences of these early-life alterations in neurotransmitter systems.

### 3.4 Lipid profiles of *Lymnaea stagnalis* organs and eggs after exposure to fluoxetine

**FIGURE 5:**
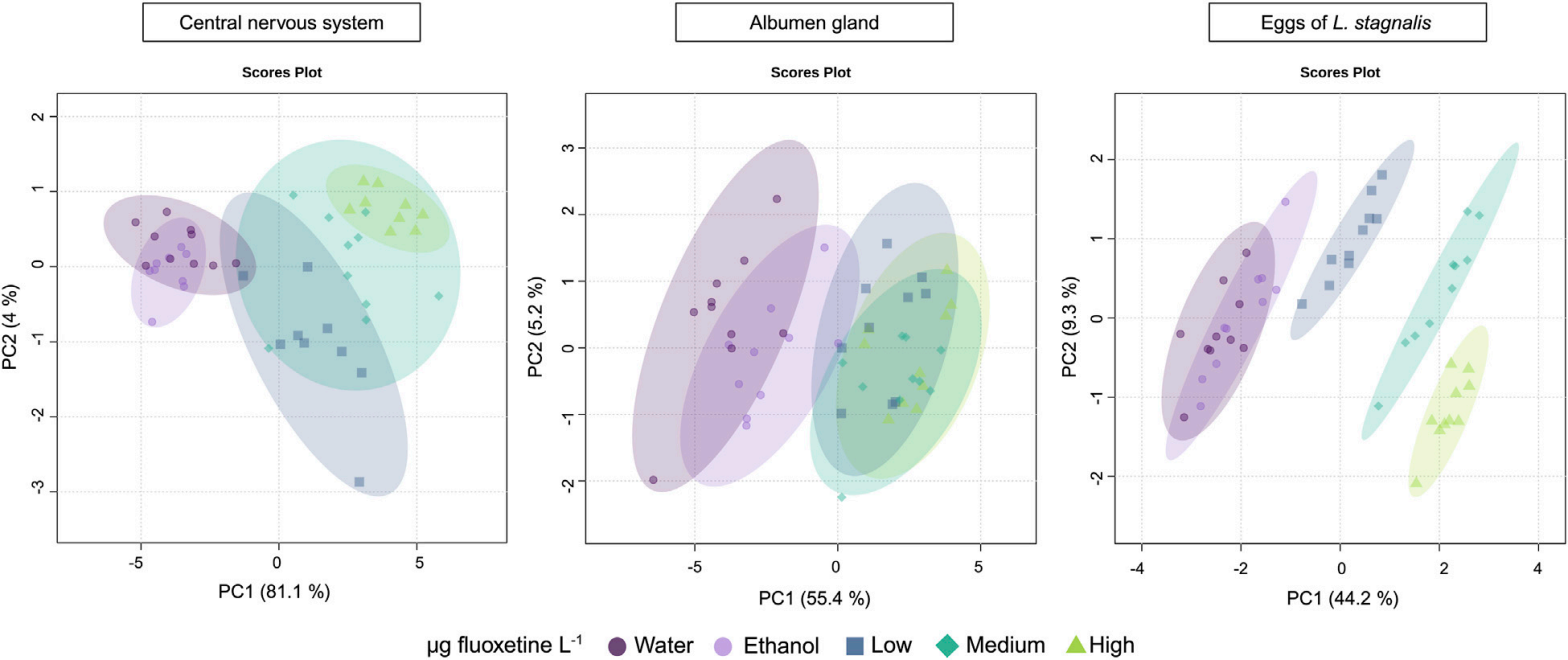
PCA score plots showing the effects of fluoxetine on the lipids of the CNS, albumen gland, and eggs of *Lymnaea stagnalis*. Fluoxetine exposure levels are categorized as µg fluoxetine L^-1^, where Low - 51 for CNS and albumen gland, 53 for eggs, Medium – 135 for CNS and albumen gland, 137 for eggs, and High - 434 for CNS and albumen gland, 417 for eggs.

Principal Component Analysis (PCA) was performed to assess the patterns in lipid profiles of *L. stagnalis* and its eggs following exposure to three concentrations of fluoxetine. For the CNS, PCA showed a substantial clustering of samples, which indicates a strong differentiation in lipid composition between the control and treated groups. The first two PC axes captured 85.1% of the total variance, indicating that these axes explain a large proportion of the variation in the data (Figure 5). In the albumen gland, the PCA showed a clustering, where groups with Medium and High fluoxetine concentrations were clustered separately from the water control group, with the first two PC axes explained 60.6% of the variance. This suggests moderate fluoxetine-induced alteration in lipid profiles compared to the CNS (Figure 5). The variance in lipid profiles of the eggs of *L. stagnalis* exposed to fluoxetine were explained by 53.5% with the first two PC axes (Figure 5). Across all three biological matrices, the High fluoxetine exposure groups tended to separate more distinctly from the controls in the PCA plots, implying a dose-dependent effect of fluoxetine on the lipidome of snails and their eggs.

**FIGURE 6:**
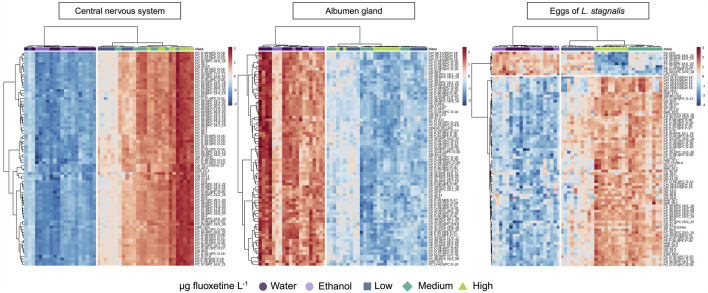
Heatmap analysis showing the effects of fluoxetine on lipids in the CNS, albumen gland, and eggs of *Lymnaea stagnalis*. The samples are clustered by treatment (Ward cluster analysis). Fluoxetine exposure levels are categorized as µg fluoxetine L^-1^, where Low - 51 for CNS and albumen gland, 53 for eggs, Medium - 135 for CNS and albumen gland, 137 for eggs, and High - 434 for CNS and albumen gland, 417 for eggs. See text for further information and Supplementary Table S5 for the lipid names.

Cluster analysis (Ward) combined with a heatmap of the lipid features of *L. stagnalis* in the CNS, albumen gland, and eggs showed the complex and tissue-specific lipidomic responses of *L. stagnalis* to fluoxetine exposure (Figure 6). In the CNS, a significant upregulation of levels of most lipids was observed, particularly at the highest fluoxetine concentrations. Based on VIP cut off (VIP >1) the most evident increase in levels was seen in diacylglycerols (DG), phosphatidylcholines (PC), and ceramides (Cer). In contrast, the albumen gland exhibited a different pattern. A general downregulation of levels of lipids was observed across all fluoxetine concentrations (Figure 6). Notably, a decrease was observed for the levels of PC and oxidized PC (PC-O), Cer, and sphingomyelins (SM).

The lipids of the eggs of *L. stagnalis* were clustered in two groups, based on the up/down-trend patterns in exposed groups compared to the control (Figure 6). Based on VIP cut off a notable decrease was observed for the levels of phosphatidylethanolamines (PE), oxidized PE (PE-O), sterols (ST) and fatty acids (FA), while PC, triacylglycerols (TG), N-acyl ethanolamines (NAE), and DG showed increased levels in exposed groups (Figure 6).

The CNS of *L. stagnalis* showed extensive lipid alterations upon fluoxetine exposure, impacting a broad spectrum of lipid classes across Low, Medium, and High concentrations. A total amount of 393 lipid features were detected, where lipid features from 21 classes were significantly affected by fluoxetine, see Table 1. Most affected lipids were Cer (17 features), DG (26 features), PC (103 features), PC-O (85 features), NAE (18 features), FA (15 features), and PE (12 features) that exhibited consistent, significant increased levels with increasing fluoxetine concentrations.

**TABLE 1:** The lipid classes in the CNS of *Lymnaea stagnalis* and the number of lipids within each class that showed a significant fold change (ANOVA, *p* < 0.05) compared to the control group after exposure to 51 (Low), 135 (Medium), and 434 (High) µg fluoxetine L^-1^. Further information can be found in Supplementary Table S5 such as the lipid acronyms.

Lipid class	Total features detected per lipid class	Total number of affected lipids (fold change, *p* < 0.05)		
		Low	Medium	High
Cer	22	17 *↑*	17*↑*	16*↑*
DG	33	26*↑*	25*↑*	26*↑*
FA	19	15*↑*	12*↑* 1*↓*	15*↑*
HexCer	2	2*↑*	2*↑*	2*↑*
LPC	11	10*↑*	10*↑*	10*↑*
NAE	22	17*↑*	17*↑*	18*↑* 1*↓*
PC	105	103*↑*	103*↑*	103*↑*
PC-O	91	84*↑*	85*↑*	85*↑*
PE	15	12*↑*	12*↑*	12*↑*
PE-O	11	9*↑*	9*↑*	9*↑*
PE P	21	19*↑*	19*↑*	19*↑*
PS	2	1*↑*	2*↑*	2*↑*
SM	4	3*↑*	3*↑*	3*↑*
ST	5	4*↑*	4*↑* 1*↓*	4*↑* 1*↓*
TG	12	9*↑*	6*↑*	3*↑*
CAR	2	2*↑*	2*↑*	2*↑*
CL	3	2*↑*	2*↑*	2*↑*
PI	2	1*↑*	1*↑*	1*↑*
SE	1	1*↑*	1*↑*	1*↑*
SG	1	1*↑*	1*↑*	1*↑*
LPE/LPE-O	4	3*↑*	3*↑*	3*↑*

The albumen gland of *L. stagnalis* exhibited pronounced lipid shifts upon exposure to fluoxetine. In contrast to the CNS, the lipid profile in the albumen gland showed extensive downward alterations upon fluoxetine exposure. A total amount of 813 lipid features were detected, where lipid features from 28 classes were significantly affected by fluoxetine, see Table 2. Lipid classes such as DG (50 significant affected features), PC (137 features), and PC-O (112 features) were heavily impacted by fluoxetine exposure. A total of 15 Cer species showed decreased levels, particularly at the High fluoxetine concentration. Additianally, NAE lipids observed to be downregulated across all concentrations with 14 significant affected features at the High concentration (Table 2).

**TABLE 2:** The lipid classes in the albumen gland of *Lymnaea stagnalis* and the number of features within each class that showed a significant fold change (ANOVA, *p* < 0.05) compared to the control group after exposure to 51 (Low), 135 (Medium), and 434 (High) µg fluoxetine L^-1^. Further information can be found in Supplementary Table S5 such as lipid acronyms.

Lipid class	Total features detected per lipid class	Total number of affected lipids (fold change, *p* < 0.05)		
		Low	Medium	High
BA	1	1*↓*	1*↓*	1*↓*
BMP	1	1*↓*		1*↓*
CAR	4	2*↓*	2*↓*	2*↓*
CE	13	7*↓*	7*↓*	8*↓*
Cer	23	15*↓*	1*↑* 14*↓*	14*↓*
CL	27	11*↓*	10*↓*	12*↓*
DG	66	47*↓*	1*↑* 44*↓*	50*↓*
FA	51	22*↓*	1*↑* 13*↓*	1*↑* 15*↓*
HexCer	2	2*↓*	2*↓*	2*↓*
LPC	24	12*↓*	7*↓*	10*↓*
LPE/LPE-O	12	5*↓*	5*↓*	5*↓*
MG	8	1*↑* 4*↓*	4*↓*	5*↓*
NAE	23	1*↑* 12*↓*	1*↑* 10*↓*	14*↓*
NAGly	6	3*↓*	3*↓*	3*↓*
PC	192	129*↓*	127*↓*	137*↓*
PC-O	155	110*↓*	1*↑* 109*↓*	112*↓*
PE	47	22*↓*	24*↓*	27*↓*
PE-O	25	11*↓*	11*↓*	11*↓*
PE P	21	21*↓*	21*↓*	21*↓*
PI		2*↓*	1*↓*	1*↓*
PS	3	3*↓*	1*↓*	2*↓*
SE	1	1*↓*	1*↓*	1*↓*
SG	1	1*↓*	1*↓*	1*↓*
SL	7	5*↓*	5*↓*	5*↓*
SM	12	8*↓*	8*↓*	9*↓*
ST	6	6*↓*	6*↓*	6*↓*
TG	65	35*↓*	31*↓*	3*↑* 38*↓*
VAE	4	2*↓*	2*↓*	3*↓*

The exposure of the eggs of *L. stagnalis* resulted in 18 significantly affected lipid classes (328 lipid featured detected in total). The total amount of detected lipid features and an overview with number of features with significant fold change (including the up/downregulation) is shown on Table 3. The pattern for lipid features increases observed in eggs exposed to fluoxetine was similar to the response of the CNS of adults *L. stagnalis*. Lipid classes as DG, PC, and PC-O were significantly affected across all exposure levels, with 25, 74, and 40 showed an increase in most lipid features feom those classes, respectively. Similarly to CNS, Cer (17 features), acylcarnitines (CAR, 3 features), and NAE (13 features) showed significant increased levels. Several lipid classes as TG, PE, PC (including PC-O), and FA showed both up- and downregulation of lipid features in exposed groups (Table 3).

**TABLE 3:** The lipid classes in the exposed eggs of *Lymnaea stagnalis* and the number of features within each class that showed a significant fold change (ANOVA, *p* < 0.05) compared to the control group after exposure to 53 (Low), 137 (Medium), and 417 (High) µg fluoxetine L^-1^. Further information can be found in Supplementary Table S5 such as lipid acronyms.

Lipid class	Total features detected per lipid class	Total number of affected lipids (fold change, *p* < 0.05)		
		Low	Medium	High
CAR	4	3*↑*	3*↑*	3*↑*
Cer	20	3*↑*	14*↑*	17*↑*
CL	3		2*↓*	2*↓*
DG	35	13*↑*	21*↑* 1*↓*	25*↑* 1*↓*
FA	15		1*↓*	1*↑* 5*↓*
HexCer	1		1*↑*	1*↑*
LPC	12	1*↑*	1*↑* 5*↓*	1*↑* 4*↓*
MG	4	1*↓*	1*↑* 1*↓*	1*↑* 1*↓*
NAE	24	8*↑*	13*↑*	13*↑*
PC	92	71*↑* 3*↓*	74*↑* 5*↓*	70*↑* 6*↓*
PC-O	52	40*↑*	40*↑* 5*↓*	37*↑* 6*↓*
PE	15	6*↑*	5*↑* 6*↓*	2*↑* 9*↓*
PE-O	7	1*↑*	2*↑* 2*↓*	4*↓*
PE P	19	17*↑*	17*↑*	14*↑* 1*↓*
SG	1	1*↑*	1*↑*	1*↑*
SL	1			1*↓*
SM	4	3*↑*	3*↑*	4*↑*
ST	5	3*↑* 1*↓*	2*↑* 1*↓*	2*↑* 1*↓*
TG	8	1*↑*	1*↑*	2*↑* 1*↓*

## 4 Discussion

### 4.1 Effect of fluoxetine on the adverse outcome and egg development

Our study demonstrates that 7-day exposure to fluoxetine did not significantly impact the survival, feeding behavior or fecundity of *L. stagnalis*. However, while we did not observe these effects, other studies have reported significant behavioral changes in aquatic species following long-term fluoxetine exposure. For instance, fluoxetine induced larval parturition and altered reproductive behavior in freshwater mussels (Bringolf et al., 2010), potentially leading to the release of underdeveloped or nonviable larvae, which could disrupt population stability. Martin et al. (2019) reported reduced anxiety-related behavior in female eastern mosquitofish after 28 days of exposure to environmentally relevant concentrations (61 and 352 ng fluoxetine L^-1^), while Wiles et al. (2020) observed increased male coercive copulatory behavior in guppies exposed for 15 months Bertram et al. (2018) found increased copulatory behavior and sperm count in mosquitofish after 30 days of exposure, and Weinberger and Klaper (2014) documented significant effects on feeding in fathead minnows. These findings suggest that long-term fluoxetine exposure can significantly impact behavior and reproduction in aquatic species, highlighting the need for future studies to explore whether prolonged exposure could also affect the feeding and fecundity of *L. stagnalis*.

In our study, fluoxetine had a pronounced effect on egg development. The hatching rate of synchronized eggs exposed to fluoxetine for 10 days was significantly reduced, with an EC50 of 126 μg/L, indicating clear dose-dependent toxicity. Additionally, eggs laid by exposed snails and continuously kept in fluoxetine-spiked water exhibited an increased ratio of non-developed and postmetamorphic embryos, suggesting impaired development. However, when eggs were transferred to clean water after being laid by exposed snails, no significant differences in hatching rates or postmetamorphic embryo ratios were observed, although the rate of non-developed eggs remained significantly higher than in the control groups.

The results of the recovery test for eggs of *L. stagnalis* indicated a possible maternal effect on embryo development. Even after transfer to clean water, the level of non-developed eggs remained significantly higher compared to controls, indicating that maternal exposure to fluoxetine may have altered the embryo development. While the EC50 observed in this study exceeds typical environmental concentrations of fluoxetine (from low ng up to 1 µg fluoxetine L^-1^), it emphasizes the potential risks in areas where pharmaceutical discharge is concentrated. Additionally, the observed developmental impairments in *L. stagnalis* eggs highlight the importance for further research into the effects of fluoxetine on reproduction and development in aquatic species, to better predict fluoxetine contamination imact on population stability over time.

The findings indicate that fluoxetine has detrimental effects on egg development during continuous exposure, while some of these effects can be partially mitigated if the eggs are removed from the contaminated environment. This suggests potential for reversing fluoxetine-induced developmental toxicity, which could inform decision-making policies. These results imply that removing fluoxetine from freshwater environments could significantly reduce population perturbations in *L. stagnalis*. To get the full picture on the populational level, the difference in responses between laboratory strains and wild populations of *L. stagnalis* needs to be addressed as well. Stress responses can differ due to genetic and environmental variation. For example, Dalesman et al. (2011) demonstrated significant differences in memory formation across geographically distinct populations of *L. stagnalis*. In wild populations, simultaneous exposure to multiple stressors, such as temperature fluctuations, heavy metals, and light pollution, may interact with fluoxetine contamination, potentially amplifying or mitigating the effects observed in controlled laboratory studies. While our study focused on a mechanistic understading of fluoxetine exposure on laboratory strains of *L. stagnalis*, future research incorporating wild populations will be crucial to evaluate whether these findings are representative of natural ecological conditions (Gasch et al., 2016; Nakadera et al., 2020).

### 4.2 Relationship between disrupted lipidome, neurotransmitters, amino acids, and phenotypic alteration

#### 4.2.1 Neurotransmitters and amino acids

Fluoxetine exposure significantly altered the neurotransmitter and amino acids profiles in the CNS, albumen gland and eggs of *L. stagnalis*, indicating its broad impact on neurochemical pathways. The CNS plays a critical role in regulating movement, feeding behavior, and development, and the observed changes in neurotransmitters such as dopamine, serotonin, and epinephrine suggest potential disruptions in these functions (Woods, 2020). For example, dopamine is involved in locomotion and neuroendocrine regulation, and its increase could lead to altered movement patterns and stress responses. Similarly, the increase in serotonin at lower fluoxetine concentrations highlights its role in regulating propagation cycles and embryonic development (Ivashkin et al., 2015; Voronezhskaya, 2021). Fluoxetine, as an SSRI, blocks the re-uptake of serotonin, resulting in a higher and longer presence of serotonin in the synaptic cleft, leading to increased postsynaptic activation. In previous study, Filla et al., 2009 described the role of two major monoaminergic systems, the dopaminergic and serotonergic, in investigating the duration of embryogenesis and movement of *L. stagnalis*. They found an effect of serotonin on the locomotor behavior of embryos. Additionally, serotonin was proposed to play a role in the execution of male reproductive behavior in hermaphroditic freshwater snails (Koene, 2010). This raises the possibility that fluoxetine-induced changes in serotonin levels could influence male sexual behavior in *L. stagnalis*, which would be an interesting area for future studies. Interestingly, in the current study, only exposure to 30 ng/mL fluoxetine affected serotonin levels in the CNS, while serotonin levels in the Medium and High exposure groups did not differ significantly from the control group. Such a non-monotonic response, with induced sensitivity at the Low concentration, has been previously observed in animal studies, suggesting that low-level stressors can trigger specific stress responses, whereas higher levels may activate different defense mechanisms and avoidance behaviors.

In the CNS, significant increases in glutamine, glutamate, and GABA were observed across all fluoxetine exposure levels compared to controls (Supplementary Figure S6). An increase in glutamine may indicate metabolic adjustments to meet elevated energy requirements triggered by fluoxetine-induced stress. Glutamine is primarily involved in protein synthesis, energy production, and the glutamine-glutamate/GABA cycle, serving as an intermediary between glutamatergic and GABAergic pathways (Walls et al., 2015). Futher, an increase in GABA levels, the primary inhibitory neurotransmitter, particularly at the High fluoxetine concentration, might indicate a compensatory mechanism to counterbalance the overall increase in neurotransmitter levels, possibly affecting locomotion and sensory processing. Additionally, it was previosly shown that disruption of amino acids reflects changes in protein composition, which can result in lower reproduction rates (Hegedüs et al., 2004).

Decreased glutamate levels in egg samples indicate altered energy metabolism and neuronal activity, primarily associated with delays in neural development and metamorphosis (Nesic et al., 1996). Significant decreases in glutamate in exposed eggs, a key neurotransmitter for synaptic plasticity and excitatory signaling, is particularly interesting as glutamate is essential for proper neural development, and its alteration may have profound effects on the neurodevelopmental trajectory of snail embryos.

Another neurotransmitter that influences embryo development, particularly metamorphosis, is histamine (Sutherby et al., 2012; Swanson et al., 2012). Histamine levels were much lower in the albumen gland of exposed snails and in the eggs. The decrease in histamine levels in the albumen gland, which provisions eggs with albumen and affects general oocyte quality (Duncan, 1958), may be interconnected with maternal influence on the success of egg development. In adult snails, lower histamine levels in pereferic organs and higher level of this animo acid in the CNS may imply impairment of processes such as neural function and reproduction, potentially reducing their ability to respond to environmental stressors or optimize reproductive output (Hegedüs et al., 2004). Furthermore, the low histamine levels in fluoxetine-exposed eggs might indicate a combined influence of the chemical on embryo development, acting both through the adults and the eggs themselves. Such similar histamine response underscores its importance as a marker for fluoxetine-induced neurochemical disruption.

The cholinergic pathway, crucial for inflammatory responses, showed significant changes, particularly with increased choline levels in the CNS. This increase suggests enhanced acetylcholinesterase (AchE) activity, leading to inflammation and potential neural degeneration, as observed in previous studies (Gnatyshyna et al., 2023; Horzmann and Freeman, 2016). The altered balance between acetylcholine and choline in eggs points to developmental disruptions, potentially delaying embryogenesis and affecting metamorphosis success (Tufi et al., 2016).

In the albumen gland, decreased levels of catecholamines like dopamine and increased breakdown products such as 3-MT, suggest disrupted catecholamine metabolism, potentially affecting reproductive processes due to the gland’s role in egg production (Koene, 2010). Notably, changes in adrenergic system, indicated by increaed normetanephrine levels, may influence the reproductive health of *L. stagnalis* (Gnatyshyna et al., 2023).

Epinephrine, a catecholamine essencial for regulating hormones, stress responses, larval development, and metamorphosis, showed an upward trend at Low and Medium fluoxetine levels in eggs. This suggests potential disruptions in neurodevelopment and metamorphosis success (Gallo et al., 2016). In the CNS, epinephrine levles rose at the High fluoxetine doses, potentially reflecting activation of the stress response system. As in other organisms, epinephrine in snail is linked to ‘fight-or-flight’ reactions, which may alter their ability to respond to environmental threats.

Catecholamines (dopamine, norepinephrine, and epinephrine) also known to influence lipid metabolism. For instance, epinephrine increases the activity of epinephrine-sensitive lipase, promoting FA release and ß-oxidation (Vaughan, 1966). Some catecholamines can also elevate TG and PC levels (O’Donnell et al., 1988). In the current study, the lipid analysis of the CNS in exposed snails revealed upregulation of FA, TG, and PC lipid features, suggesting that the stress response of *L. stagnalis*, observed through neurotransmitter and amino acid analysis, is interconnected with lipid disruptions. Similarly, exposed eggs exhibited the same interconnection, although the upregulation of FA and TG lipids was much less pronounced.

Phenylalanine, a precursor of dopamine and epinephrine, plays a critical role in neural function, and imbalances in phenylalanine/dopamine levels are associated with neurological disorders (Wyse et al., 2021). Elevated levels of phenylalanine can disturb amino acid transport to the brain, impair synthesis of serotonin, and proteins, induce inflammation and impairing neural signaling (Wyse et al., 2021). In the current study, fluoxetine exposure appears to disrupt catecholaminergic pathways by affecting phenylalanine metabolism. The observed increases in phenylalanine levels, along with elevated levels of l-tyrosine, dopamine, and epinephrine, suggest that fluoxetine affects the entire tyrosine pathway in the CNS. Since l-tyrosine is directly converted into dopamine, changes in its levels may influence the reward and motivation systems in the snails (Brown et al., 2018). Furthermore, increased l-tyrosine levels in the exposed eggs of *L. stagnalis* could indicate an adaptive response aimed at maintaining developmental processes despite fluoxetine exposure.

Fluoxetine exposure significantly altered neurotransmitter and amino acid profiles in *L. stagnalis*, indicating disruptions in key neurochemical pathways. Specifically, the modulation of serotonin, dopamine, choline, and epinephrine levels suggests that fluoxetine induces stress responses, inflammation, and metabolic shifts that could impair neural signaling and developmental processes. These biochemical disruptions could lead to decreased reproduction rates and impaired embryonic development, ultimately affecting the population dynamics of *L. stagnalis*. Further research is necessary to understand the full impact of fluoxetine on aquatic invertebrate populations and to develop strategies for mitigating these effects in natural environments.

#### 4.2.2 Lipid profile changes

Fluoxetine exposure induced significant lipid alterations in the CNS, albumen gland, and eggs of *L. stagnalis*, showing its impact on lipid metabolism and membrane integrity.

In the CNS, nearly every annotated lipid class had one or more lipids affected by fluoxetine, suggesting that the CNS was the most sensitive to fluoxetine, resulting in widespread lipid changes. Cer, a class of sphingolipids, play a significant role in brain development, including tissue development and cell recognition. Cer lipids are crucial for the myelination of axons (Dasgupta and Ray, 2017). The upregulation of Cer could indicate increased brain protection activity, and therefore a possible inflammatory response in *L. stagnalis*. Cer lipids also function as signaling molecules, influencing the dopamine and serotonin transporter’s function and their substantial upregulation in the CNS of exposed snails potentially reflects disruptions in neural cell function and monoamine balance (Bartke and Hannun, 2009; Riddle et al., 2003). The upregulation of both dopamine and Cer can be an indicator of wide influence of fluoxetine on the CNS of invertebrates, well beyond serotonin pathway.

Another lipid cluster, fatty acyls (including CAR, FA and NAE), showed significant alterations in the CNS. A recent study on lipidomics in *L. stagnalis* reported the downregulation of many FA, indicating neuron cell injury (Tufi et al., 2015). In contrast, the current study observed an increase in FA levels in the CNS of exposed snails, along with elevated levels of CAR. CAR lipids are involved in FA transport and levels of PC (Bouly et al., 2022). The increase in the fatty acyl pool in exposed snails is a clear indication of cellular stress and energy imbalance in the CNS (Han, 2016). Endocannabinoids, or NAE, also showed an upward trend in the CNS. The production of NAE involves the transformation of Acetyl-CoA and FA and plays a crucial role in lipid peroxidation and energy metabolism. The high levels of altered FA and NAE, together with PC-O and PE-O, might indicate lipid peroxidation and further membrane imbalance in the CNS caused by fluoxetine exposure (Han, 2016).

Additionally, glycerolipids such as TG and DG showed an upward trend in the CNS of exposed snails. The transformation pathway between TG and DG, with FA as a product of conversion, is essential for energy metabolism, vitellogenesis, and egg mass formation. Furthermore, the synthesis and catabolism of TG are highly dependent on FA. The strong increase in TG, DG, and FA indicates disturbances in glycerolipids transformation pathways, suggesting energy disruption due to fluoxetine exposure (Alves-Bezerra et al., 2016; Dunning et al., 2014).

Changes in the lipid profile of the albumen gland in exposed animals often exhibited an opposite direction compared to the CNS. The downregulation of lipid classes, including PC, PC-O, and SM, suggests suppressed lipid synthesis or increased catabolism, possibly reflecting stress-induced metabolic alterations. These changes could impair the gland’s functionality in egg production and overall reproductive health (Martínez-Pita et al., 2012; Nagle et al., 1999). The decrease in FA and CAR levels, essential for beta-oxidation and oocyte quality, further indicates disturbances in egg production (Dunning et al., 2014). Additionally, reduced NAE levels point to disruptions in energy metabolism and anti-inflammatory responses in the albumen gland (Han, 2016).

A decrease in glycerolipids, specifically TG and DG, was also observed in the albumen gland of exposed snails. Since both lipid classes are essential for energy storage and reproductive activity, the maturation and quality of oocytes, along with the available energy in the albumen gland, may have been negatively affected or reduced by fluoxetine exposure (Alves-Bezerra et al., 2016; Dunning et al., 2014). The overall decrease in lipid metabolite abundance in the albumen gland, in contrast to the CNS, highlights the tissue-specific effects of fluoxetine in *L. stagnalis*. Notably, the decrese in essential lipid in albumen gland may play a role in the maternal effect that we observed in egg recovery test, with the rate of non-developed embryos remained significantly higher from exposed snails.

In exposed eggs, the mixed response in lipid profiles, with both upregulation and downregulation of various lipid species, indicates a complex impact of fluoxetine on embryonic development. The observed increases in PC, PC-O, and DG suggest enhanced lipid metabolic activity, whereas decreases in some PE, PE-O, sterols (ST), and FA lipid features highlight potential disruptions in energy metabolism and membrane integrity, considering the especially the role of PE and PE-O in mitochondrial respiration and lipid homeostasis (Patel and Witt, 2017). These alterations could impact embryonic growth, developmental outcomes, and long-term viability (Sun et al., 2023; Tufi et al., 2016).

The sphingolipids of the exposed eggs of *L. stagnalis* have followed the same pattern as the lipid features of CNS. Notable, an increase of Cer species was observed at all exposure concentrations. Previously, the changes of Cer metabolism were assessed through gene expression analysis on marine mussel *Mytilus galloprovincialis*, where Cer levels were considered to be an important modulator in early developmental stages (Balbi et al., 2023). Additionally, the role of ceramides and the enzymes involved in their metabolism was assessed in *Caenorhabditis elegans*. The study highlighted the essential role of ceramide metabolism in embryotic cell division and oocyte formation (Nomura et al., 2011). The dysregulation of Cer in both eggs and the CNS of *L. stagnalis* shows the impact of fluoxetine on the reproductive system of adult snails and possible influence on embryo development.

Another lipid class that closely related with early stages of embryo development is CAR. The alterations of CAR llipids could influence the possibility for oocytes to reach the blastocyst stage (Dunning et al., 2014). The observed increase in NAE, associated with inflammation processes and in energy metabolism, further indicates stress-induced matabolic disturbances in embryos (Han, 2016). The high amount of altered PC-O and PE-O, might indicate lipid peroxidation and further membrane imbalance in eggs.

The analysis of lipid changes in eggs revealed similarities with both the CNS and the albumen gland of *L. stagnalis*. Interestingly, some lipid classes in exposed eggs, such as FA, exhibited responses resembling those observed in the albumen gland of exposed snails. Conversely, the lipid responses of TG, DG, NAE, Cer, and CAR were more comparable between the eggs and the CNS. Additionally, in exposed eggs, both PC and PE (including their oxidized forms) demonstrated fold changes that were both upregulated and downregulated. These changes mirrored the downregulation of phospholipids observed in the albumen gland and the upregulation seen in the CNS. These patterns highlight lipid classes such as TG, DG, NAE, Cer, CAR, PC, and PE as particularly interesting candidates for future studies, which could further explore their roles as biomarkers and their relevance to fluoxetine-induced lipidomic disruptions in *L. stagnalis*.

#### 4.2.3 Linking choline changes to PC and oxidized PC lipids

Choline is a key precursor for the synthesis of PC, a major component of cell membranes. The CDP-choline pathway (Kennedy pathway) is the primary route for PC synthesis in most tissues (McMaster, 2018). In this pathway, choline is phosphorylated to phosphocholine by choline kinase, then converted to cytidine diphosphate-choline (CDP-choline) by CTP-cytidylyltransferase, and finally, CDP-choline is combined with DG to form PC. The observed increase in choline levels in the CNS and eggs following fluoxetine exposure could indicate an upregulation of the initial steps of the CDP-choline pathway. This might represent a compensatory mechanism to maintain or increase PC synthesis under stress conditions induced by fluoxetine. PC lipids are susceptible to oxidation, especially under oxidative stress conditions, and PC-O lipids serve as markers of lipid peroxidation and cellular oxidative damage (Han, 2016). The increase in PC, PE, PC-O, and PE-O levels observed in the CNS and eggs suggests that fluoxetine exposure induces oxidative stress. This oxidative modification of PC and PE lipids can alter membrane properties, affecting fluidity, permeability, and membrane associated enzyme activities, potentially disrupting cellular functions. The changes in choline, PC, and PC-O metabolism could contribute to the neurochemical and behavioral alterations observed in *L. stagnalis* following fluoxetine exposure. The connection observed between these metabolites offers a view of the effects of SSRI pharmaceuticals on aquatic invertebrates, bridging molecular observations with physiological and behavioral outcomes. Further investigation of these links may be an important step in integrating omics analysis into classical risk assessment and ecotoxicology.

## 5 Conclusion

This study provides insight into the impact of fluoxetine exposure on *L. stagnalis*, with a focus on egg development, neurochemical pathways, and lipid metabolism. While 7-day exposure to fluoxetine did not significantly affect survival, feeding behavior, or fecundity, it notably impaired egg development. Fluoxetine-induced developmental toxicity was dose-dependent, with a clear reduction in hatching rates and an increased proportion of non-developed embryos, especially when eggs were continuously exposed. While the concentrations tested in this study were higher than fluoxetine levels typically detected in the environment, the observed effects highlight the potential for developmental toxicity under conditions of pharmaceutical accumulation in aquatic ecosystems. This study also demonstrated, for the first time, a potential maternal effect of fluoxetine on embryo development. Additionally, a recovery test was conducted to assess the possibility of population recovery if fluoxetine were removed from the environment. Importantly, removing eggs from the contaminated environment partially mitigated the developmental damage, suggesting that only partial recovery is possible if the pollutant is removed from the ecosystem.

In this study, the findings emphasize the value of integrating behavioral assessments with molecular analysis to improve mechanistic understanding of adverse outcomes in classical risk assessments. For example, we proposed links between neurotransmitter, amino acid, and lipid alterations and embryo development, feeding and reproduction in *L. stagnalis*. Fluoxetine exposure resulted in significant alterations in the metabolome of the CNS of adults, which were further reflected in the eggs (e.g., increases in catecholamines, Cer, PC, PC-O, and choline). Additionally, molecular changes in the albumen gland of exposed snails (e.g., decreases in histamine and FA) were similarly mirrored in the metabolic profile of their eggs, indicating systemic effects.

Although this 1-week exposure study provides some insights, the long-term effects of fluoxetine on adult behavior should be further investigated. Long-term exposure studies of fluoxetine impact together with recovery assessment will be critical for informing water treatment strategies and improving ecological risk assessments for aquatic species.

## Data Availability

The datasets presented in this study can be found in online repositories. The names of the repository/repositories and accession number(s) can be found below: https://doi.org/10.5281/zenodo.14235510.

## Glossary

ACN: acetonitrile
ANOVA: analysis of variance
BA: bile acids
BMC: benchmark concentrations
BMP: bismonoacylglycerophosphates
BPUs: benzoylphenylureas
CAR: acylcarnitines
CE: cholesteryl esters
Cer: ceramides
CL: cardiolipins
CNS: central nervous system
DG: diacylglycerols
ESI: electrospray ionization
EtOH: ethanol
FA: fatty acids
FDR: false discovery rate
HexCer: neutral glycosphingolipids
HPLC: high-performance liquid chromatography
IPA: 2-propanol
IS: internal standards
LPC: lysophosphatidylcholines
LPE: lysophosphatidylethanolamines
MeOH: methanol
MG: monoacylglycerols
MUFA: monounsaturated fatty acids
MZML: mass spectrometry markup language
NAE: N-acyl ethanolamines
NAE 16:0: N-palmitoylethanolamine
NAE 18:0: N-stearoylethanolamine
NAGly: N-acyl glycines
PCA: principal component analysis
PC: phosphatidylcholines
PE: phosphatidylethanolamines
PI: phosphatidylinositols
PLS-DA: partial least squares-discriminant analysis
PS: phosphatidylserines
QC: quality control
QTOF: quadrupole time-of-flight
ROS: radical reactive species
SE: sterol esters
SL: sulfonolipids
SM: sphingomyelins
ST: sterol lipids
TG: triacylglycerols.
