# Supplementary material for: Impact of fluoxetine exposure on Lymnaea stagnalis and its developing eggs: integrating untargeted lipidomics, targeted metabolomics, and classical risk assessment
Source: Front Pharmacol. 2025 Feb 4;16:1536438. doi: 10.3389/fphar.2025.1536438 (PMC11832466; doi:10.3389/fphar.2025.1536438)
Supplement: Supplementary file 1 [file DataSheet1.docx]

**Supporting information for manuscript:** Impact of Fluoxetine Exposure on *Lymnaea stagnalis* and Its Developing Eggs: Integrating Untargeted Lipidomics, Targeted Metabolomics, and Classical Risk Assessment

Material and Methods

Figure S1. Stages of *L. stagnalis* egg development. Stages are presented with representative images of egg masses (top panel) and individual eggs (bottom panel) for each category: Developed eggs (corresponding to 10 days of embryogenesis, purple), Postmetamorphic stage (equivalent to seven days of embryogenesis, blue), Non-developed - Veliger stage (equivalent to three to four days of development, dark green), and Non-developed embryos (green). The classification was conducted using ImageJ software to facilitate developmental assessment.
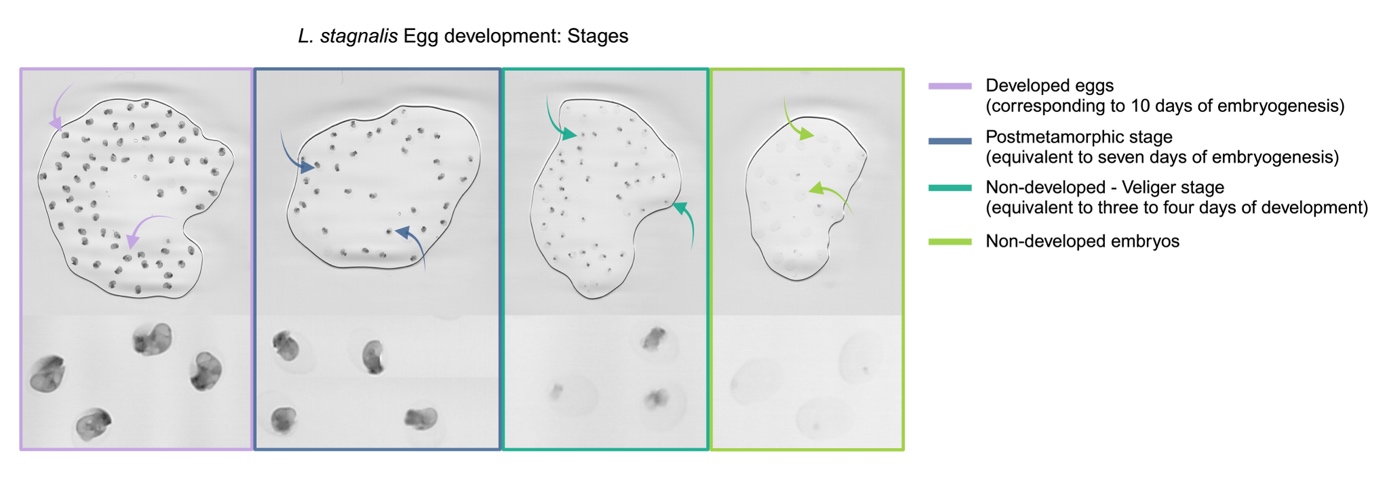


Table S1. Neurotransmitters and amino acids, and their isotope-labeled standards general information: provider, retention time (RT), and mass transitions.

| Neurotransmitters and animo acids and isotope-labeled standards ID | Provider | RT, min | Detected Mass | Quantification trace 1 | Quantification trace 2 |
| --- | --- | --- | --- | --- | --- |
| 3-MT | Sigma Aldrich | 2.00 | 168.3 | 151.1 | 91.1 |
| 3-MT(D4) | Sigma Aldrich | 2.00 | 172.1 | 155.1 | 95.2 |
| Acetylcholine | Sigma Aldrich | 1.68 | 146.2 | 87.1 | 43.3 |
| Acetylcholine:HCL(D4) | Cambridge Isotope Laboratories | 1.66 | 150.3 | 91.2 | 43.3 |
| Choline | Sigma Aldrich | 2.43 | 104.2 | 60.3 | 45.3 |
| Choline(D3) | Sigma Aldrich | 2.43 | 117.3 | 69.3 | 49.3 |
| Dopamine | Sigma Aldrich | 2.70 | 154.1 | 137.1 | 91.1 |
| Dopamine:HCL(D4) | Sigma Aldrich | 2.70 | 158.2 | 141.1 | 95.2 |
| Epinephrine | Sigma Aldrich | 3.24 | 166.1 | 77.2 |  |
| DL-Epinephrine (1,2-13C2, 99%; 15N, 98%) | Cambridge Isotope Laboratories | 3.05 | 187.2 | 169.1 |  |
| γ-Aminobutyric acid (GABA ) | Sigma Aldrich | 3.57 | 104.2 | 87.1 |  |
| 4-Aminobutyric acid-2,2,3,3,4,4-d6 | Sigma Aldrich | 3.57 | 110.1 | 93.2 |  |
| Glutamate | Sigma Aldrich | 7.75 | 148.1 | 84.2 | 130.1 |
| Glutamine | Sigma Aldrich | 8.13 | 147.0 | 84.1 | 130.1 |
| Histamine | Sigma Aldrich | 8.60 | 112.2 | 95.1 | 68.2 |
| Histamine:2HCL (A,A,B,B-D4, 98%) | Cambridge Isotope Laboratories | 8.60 | 116.0 | 99.0 |  |
| Histidine | Sigma Aldrich | 9.75 | 156.2 | 110.1 |  |
| Norepinephrine | Sigma Aldrich | 4.10 | 152.2 | 107.1 |  |
| (±)-Norepinephrine:HCL (D6, 98%) | Cambridge Isotope Laboratories | 4.11 | 176.2 | 158.1 |  |
| Normetanephrine | Sigma Aldrich | 2.66 | 166.1 | 134.1 | 121.1 |
| Phenylalanine | Sigma Aldrich | 3.25 | 166.2 | 120.1 | 103.1 |
| Serotonin-α,α,β,β,-d4 creatinine sulfate monohydrate | Sigma Aldrich | 2.25 | 181.2 | 164.1 | 118.2 |
| Serotonin | Sigma Aldrich | 2.25 | 177.0 | 166.0 |  |
| Tryptophan | Sigma Aldrich | 3.25 | 205.0 | 188.1 | 146.1 |
| Tryptophan-d3 | Sigma Aldrich | 3.25 | 208.2 | 191.1 | 147.1 |
| Tyramine | Sigma Aldrich | 2.05 | 138.1 | 121.1 | 77.2 |
| Tyramine:HCL (1,1,2,2-D4, 98%) | Cambridge Isotope Laboratories | 2.05 | 142.0 | 125.0 |  |
| Tyrosine | Sigma Aldrich | 4.97 | 182.1 | 136.2 | 165.2 |
| Tyrosine-d4 | Sigma Aldrich | 5.00 | 186.0 | 140.2 | 169.1 |

Table S2. Neurotransmitters and amino acids quantification details: limit of detection (LOD) and limit of quantification (LOQ), together with the linearity of the calibration curve for the methods described in the current study. LOD and LOQ were establishes by the signal to noise ratio (S/N) 3 and 10, respectively. All LOD and LOQ values are presented in ng fluoxetine mL^-1^.

| Neurotransmitters | LOD | LOQ | R^2^ |
| --- | --- | --- | --- |
| Eggs |  |  |  |
| Choline | 0.6 | 1.9 | 0.998 |
| Acetylcholine | 0.4 | 1.2 | 0.999 |
| Glutamate | 6.7 | 22 | 0.998 |
| Epinephrine | 30 | 99 | 0.999 |
| Tryptophan | 7.0 | 24 | 0.999 |
| Tyrosine | 72 | 79 | 0.998 |
| Phenylalanine | 6.0 | 20 | 0.997 |
| Glutamine | 4.0 | 13 | 0.999 |
| Histamine | 0.4 | 1.2 | 0.998 |
| GABA | 1.5 | 5.1 | 0.999 |
| Histidine | 28 | 99 | 0.998 |
| CNS |  |  |  |
| Choline | 4.8 | 5.3 | 0.998 |
| Acetylcholine | 0.4 | 1.2 | 0.999 |
| Glutamate | 368 | 447 | 0.994 |
| Dopamine | 21 | 71 | 0.997 |
| Serotonin | 30 | 36 | 0.999 |
| Epinephrine | 28 | 93 | 0.998 |
| Tryptophan | 8.3 | 28 | 0.990 |
| Tyrosine | 315 | 353 | 0.996 |
| Phenylalanine | 8.1 | 27 | 0.99 |
| Glutamine | 14 | 46 | 0.998 |
| Histamine | 10 | 11 | 0.995 |
| GABA | 2.4 | 8.1 | 0.998 |
| Histidine | 50 | 168 | 0.996 |
| Albumen gland |  |  |  |
| Choline | 0.1 | 0.2 | 0.992 |
| Acetylcholine | 0.01 | 0.02 | 0.991 |
| Glutamate | 0.5 | 1.6 | 0.984 |
| Dopamine | 0.2 | 0.8 | 0.992 |
| Serotonin | 0.06 | 0.2 | 0.999 |
| Epinephrine | 0.6 | 2.1 | 0.997 |
| Tryptophan | 0.7 | 2.4 | 0.996 |
| Tyrosine | 3.2 | 10 | 0.989 |
| Phenylalanine | 9.7 | 32 | 0.992 |
| Glutamine | 0.8 | 2.3 | 0.994 |
| Histamine | 0.01 | 0.04 | 0.992 |
| GABA | 0.1 | 0.5 | 0.998 |
| Histidine | 0.5 | 1.6 | 0.978 |
| Normetanephrine | 0.01 | 0.05 | 0.995 |
| Norepinephrine | 1.5 | 1.7 | 0.998 |
| Tyramine | 0.04 | 0.1 | 0.992 |
| 3-MTMet | 1.2 | 1.3 | 0.998 |

Table S3. Quantification of fluoxetine in water. The measurements were performed for spiking solution (spike), and after 48 hours before change of water for both *L. stagnalis* and its eggs exposure experiments (eggs). Sampling was performed randomly for each type of samples. The final concentration of fluoxetine in exposure groups was calculated as an average between the measurements, separately for *L. stagnalis* exposure (spike and snail groups) and for egg exposure tests (spike and eggs groups). Nominal concentrations were following: Water and Ethanol (EtOH) controls – 0, Low – 30, Medium – 100, High – 300 ng fluoxetine mL^-1^.

| Treatment ID | Experiment and spike | Time of sampling, h | Actual concentration, ng fluoxetine mL^-1^ |
| --- | --- | --- | --- |
| Water | Spike | 0 | 0.0 |
| Water | Spike | 0 | 4.8 |
| Water | Spike | 0 | 0.4 |
| Water | Spike | 0 | 1.6 |
| Water | Snails | 48 | 1.9 |
| Water | Snails | 48 | 0.9 |
| Water | Snails | 48 | 8.9 |
| Water | Eggs | 48 | 0.0 |
| Water | Eggs | 48 | 0.0 |
| Low | Spike | 0 | 40 |
| Low | Spike | 0 | 56 |
| Low | Spike | 0 | 65 |
| Low | Spike | 0 | 69 |
| Low | Spike | 0 | 61 |
| Low | Spike | 0 | 67 |
| Low | Snails | 48 | 27 |
| Low | Snails | 48 | 30 |
| Low | Snails | 48 | 46 |
| Low | Eggs | 48 | 35 |
| Low | Eggs | 48 | 48 |
| Low | Eggs | 48 | 50 |
| Low | Eggs | 48 | 36 |
| Low | Eggs | 48 | 52 |
| Medium | Spike | 0 | 101 |
| Medium | Spike | 0 | 145 |
| Medium | Spike | 0 | 155 |
| Medium | Spike | 0 | 164 |
| Medium | Spike | 0 | 180 |
| Medium | Spike | 0 | 153 |
| Medium | Snails | 48 | 65 |
| Medium | Snails | 48 | 138 |
| Medium | Snails | 48 | 113 |
| Medium | Eggs | 48 | 82 |
| Medium | Eggs | 48 | 138 |
| Medium | Eggs | 48 | 136 |
| Medium | Eggs | 48 | 117 |
| High | Spike | 0 | 527 |
| High | Spike | 0 | 479 |
| High | Spike | 0 | 413 |
| High | Spike | 0 | 513 |
| High | Spike | 0 | 477 |
| High | Spike | 0 | 312 |
| High | Snails | 48 | 430 |
| High | Snails | 48 | 365 |
| High | Snails | 48 | 387 |
| High | Eggs | 48 | 321 |
| High | Eggs | 48 | 442 |
| High | Eggs | 48 | 272 |
| Average concertation of fluoxetine for assessment | Experiment | Average concentration (ng a.s. mL^-1^) | SD (n = number of replicates measured on 0 and 48h) |
| Water | Snails | 2.6 | 1.9 |
| Low | Snails | 51 | 15 |
| Medium | Snails | 135 | 34 |
| High | Snails | 434 | 67 |
| Water | Eggs | 1.1 | 1.7 |
| Low | Eggs | 53 | 12 |
| Medium | Eggs | 137 | 29 |
| High | Eggs | 417 | 88 |

Table S4. MS-DIAL parameters for peak deconvolution, peak alignment, and feature identification of lipids detected in organs and eggs of the snail *L. stagnalis*. RT- stands for Retention time. All the other setting in the software was left as standard preset.

| Analysis parameter settings | Values used |  |
| --- | --- | --- |
| **Data collection** |  | |
| MS1 tolerance | 0.1 | |
| MS2 tolerance | 0.05 | |
| RT window | 0.6 - 25 min | |
| MS 1 range | 60 - 2000 | |
| MS/MS range | 60 - 2000 | |
| **Peak detection** |  | |
| Minimum peak height | 700 | |
| Minimum peak width | 5 scans | |
| **MS2Dec** |  | |
| Sigma window value | 0.5 | |
| **Identification** |  | |
| RT tolerance | 100 min | |
| Accurate mass tolerance (MS1) | 0.01 | |
| Accurate mass tolerance (MS2) | 0.05 | |
| Identification score cutoff | 80 | |
| **Adduct** |  | |
| Positive mode | [M+H], [M+NH4], [M+Na], [M+k], [M+H-H20] | |
| Negative mode | [M-H], [M-H2O-H], [M+Na-2H], [M+Cl], [M+K-2H], [M+CH3OH-H] | |
| **Alignment** |  | |
| RT tolerance | 0.3 | |
| MS1 tolerance | 0.05 | |

Results

Feeding, reproduction, and egg development

Figure S2. Feeding behavior of *L. stagnalis* assessed by the remaining area of lettuce. The data are presented as the average lettuce area consumed each day for each exposure and control group (top panel), and as an individual assessment of feeding changes for each snail over time (bottom panel). Concentration levels are Water and Eithanol – 0, Low – 51, Medium – 135, and High – 434 ng Fluoxetine mL^-1^.


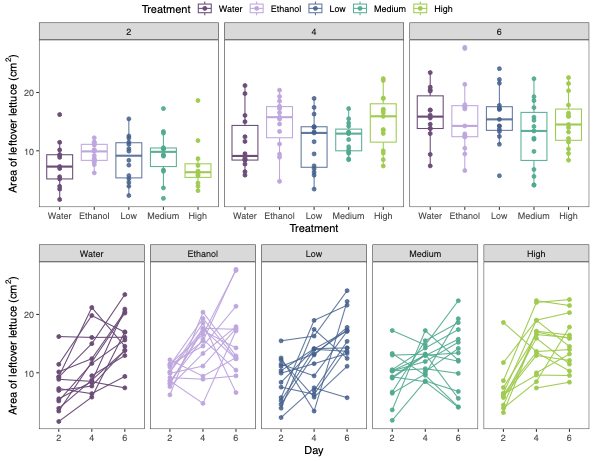


Figure S3. Fecundity, measured as the total number of eggs produced by *L. stagnalis* in each fluoxetine exposure and control group during the experiment. Concentration levels are Water and Eithanol – 0, Low – 51, Medium – 135, and High – 434 ng Fluoxetine mL^-1^.


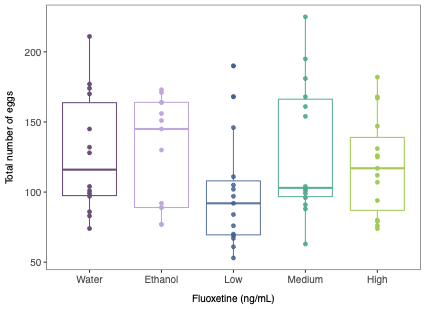


Figure S4. The effect of fluoxetine on egg development. The figure represents the development stage of the eggs laid by exposed snails and kept incubated in spiked water on days 3, 5, and 7. We had three egg samplings through the exposure (Day 3, 5, 7). We pooled the two controls (water and ethanol), concentration levels are Low – 53, Medium – 137, and High – 417 ng Fluoxetine mL^-1^.


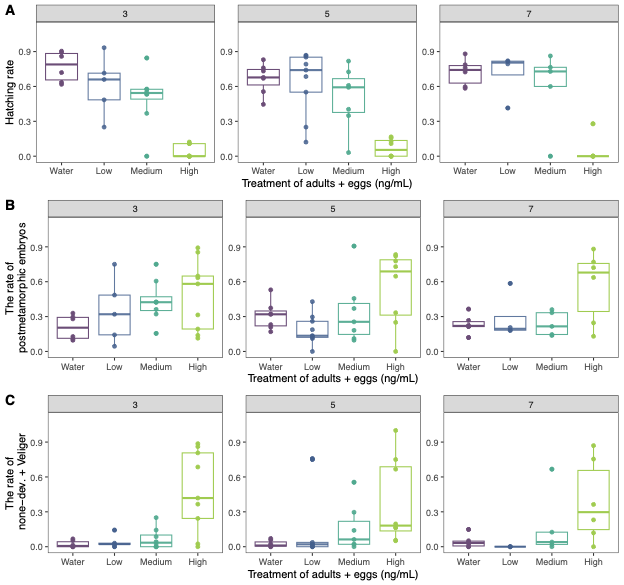


Figure S5. The recovery effect of the *L. stagnalis* eggs. The figure represents the development stage of the eggs laid by exposed snails, that were further transferred and kept incubated in water on days 3, 5, and 7 of experiment. Treatment concentration levels of adult snails are Low - 51, Medium - 135, and High - 434 ng fluoxetine mL^-1^.


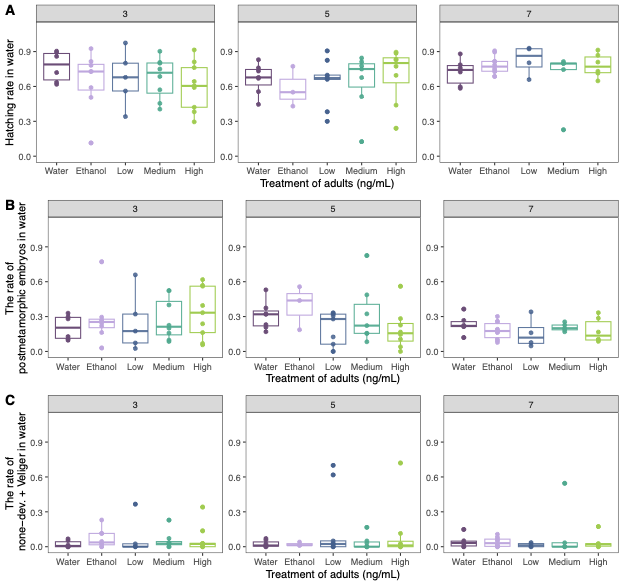


Metabolomic changes in the neurotransmitter and amino acid profiles of *L. stagnalis* organs and eggs after exposure to fluoxetine

Figure S6. Significantly affected neurotransmitters and animo acids in the central nervous system (CNS), of *L. stagnalis* exposed to fluoxetine. The concentration levels are Low - 51, Medium - 135, and High - 434 µg fluoxetine L^-1^. Significant differences (p < 0.05) between treatments are indicated by differing letters, where “a” is significance against the water control and “b” – significance against the ethanol (EtOH) control.


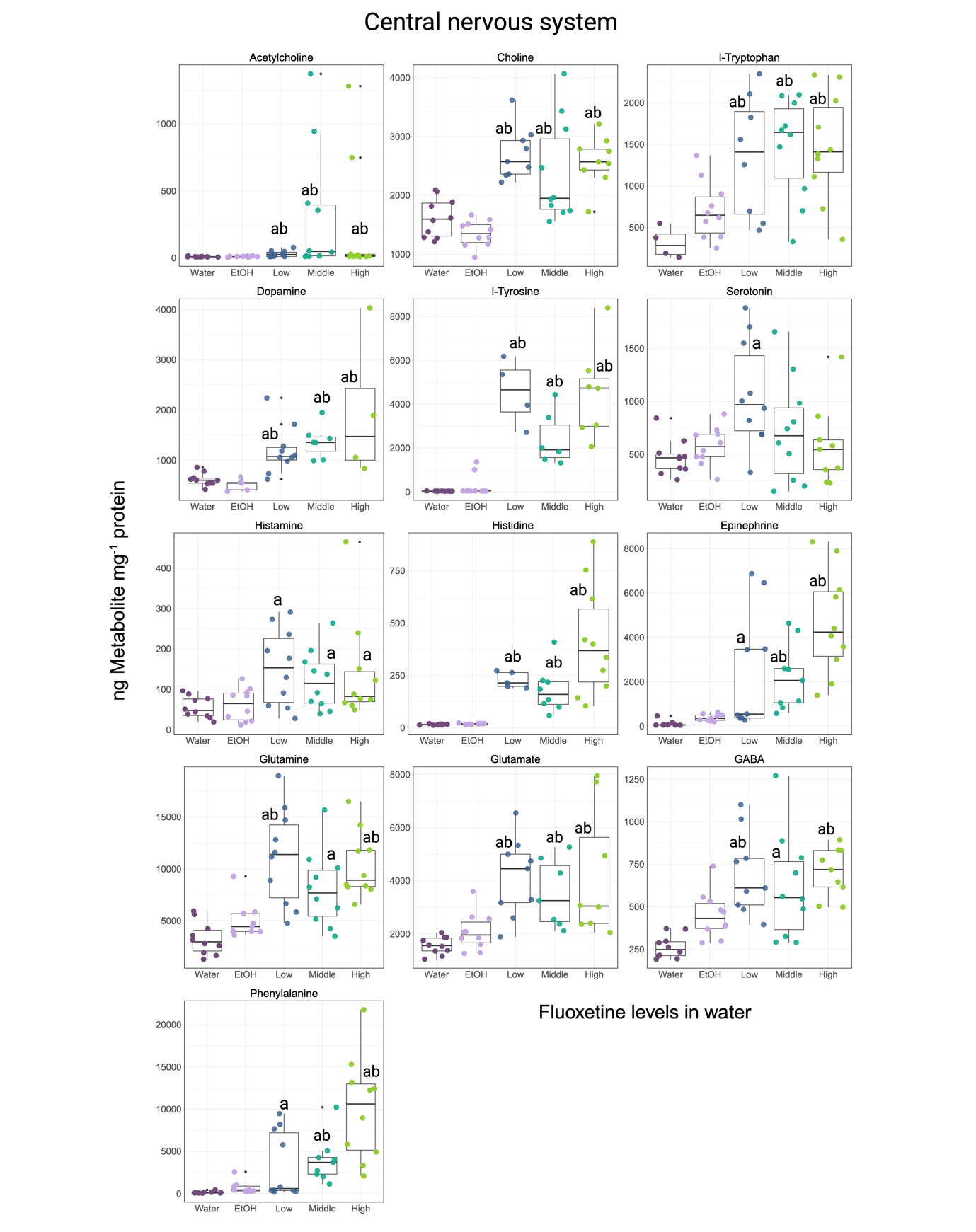


Figure S7. Significantly affected neurotransmitters and animo acids in the albumen gland of *L. stagnalis* exposed to fluoxetine. The concentration levels are Low - 51, Medium - 135, and High - 434 µg fluoxetine L^-1^. Significant differences (p < 0.05) between treatments are indicated by differing letters, where “a” is significance against the water control and “b” – significance against the ethanol (EtOH) control.


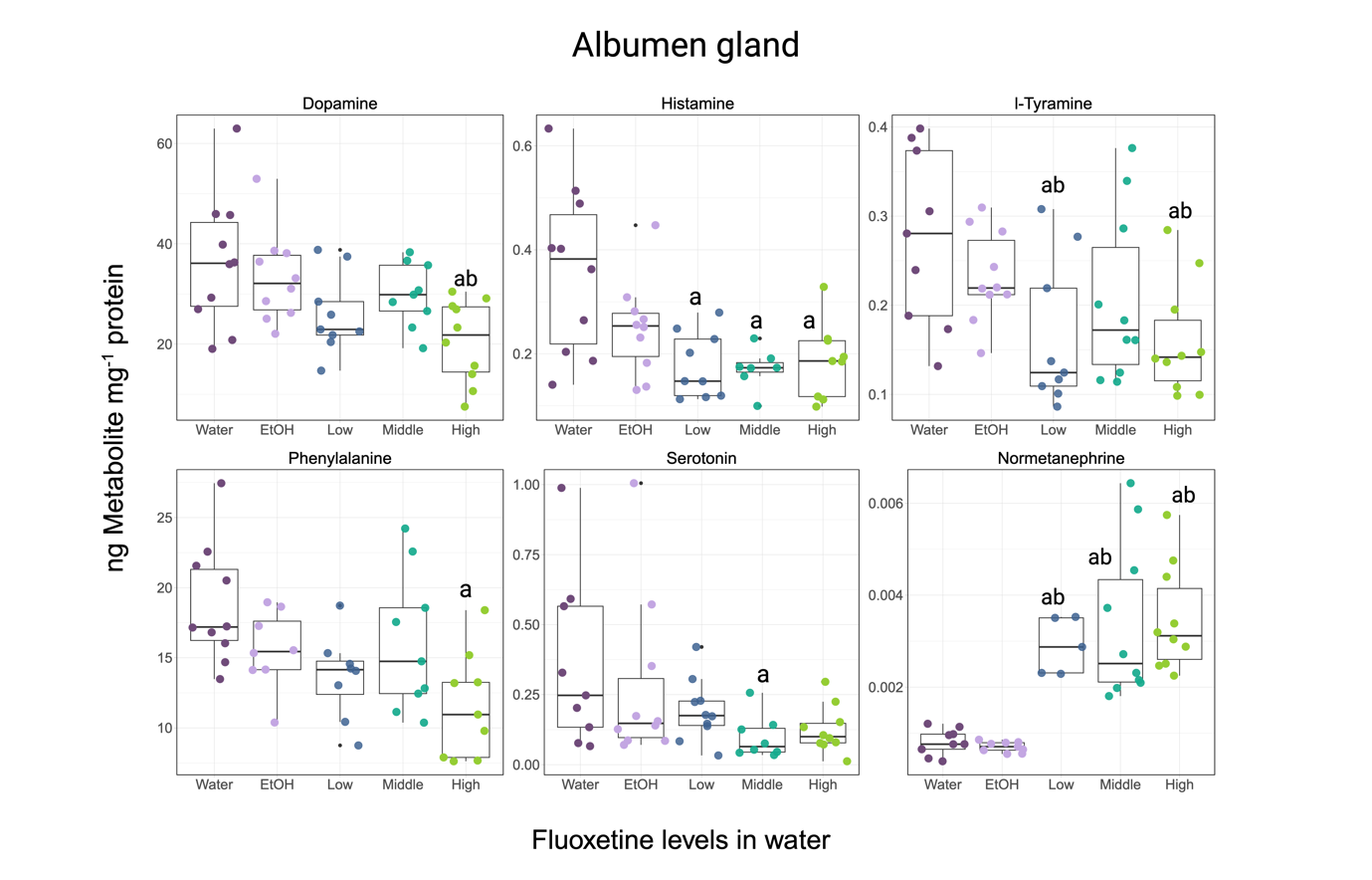


Figure S8. Significantly affected neurotransmitters and amino acids in the eggs of *L. stagnalis* exposed to fluoxetine. The concentration levels are Low - 53, Medium -137, and High - 417 µg fluoxetine L^-1^, respectively. Significant differences (p < 0.05) between treatments are indicated by differing letters, where “a” is significance against the water control and “b” – significance against the ethanol (EtOH) control.


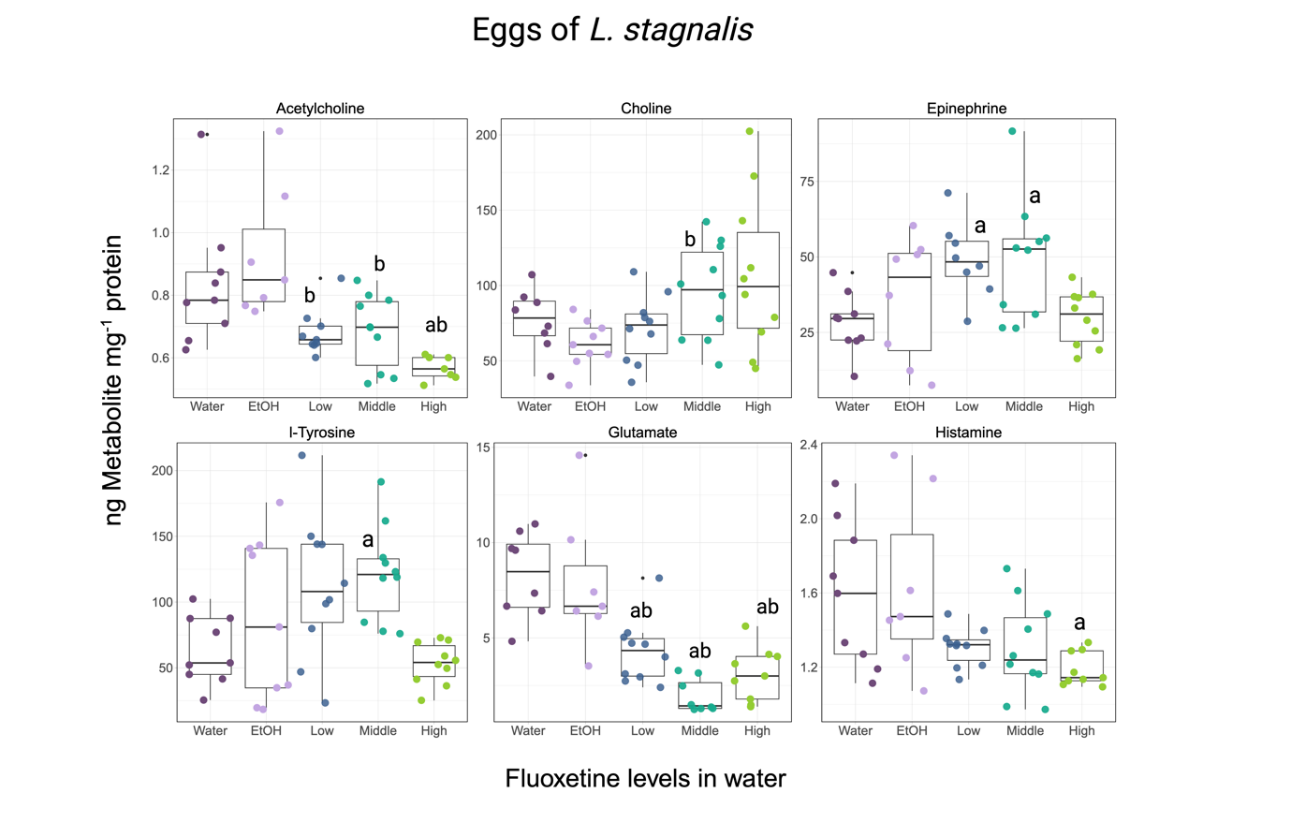


Lipid profiles of *L. stagnalis* organs and eggs after exposure to fluoxetine

Table S5. Lipid full names with abbreviations and lipid classification based on LipidBlast database.

| **Categories** | **Class** | **Abbreviation** |
| --- | --- | --- |
| Fatty acyls | N-acylethanolamines | NAE |
|  | N-acyl glycine | NAGly |
|  | Acylcarnitine | CAR |
|  | Fatty acid ester of hydroxyl fatty acid | FAHFA |
|  | Free fatty acid | FA |
| Glycerolipids | Diacylglycerol | DG |
|  | Monoacylglycerol | MG |
|  | Triacylglycerol | TG |
| Glycerophospholipids | Lysophosphatidylcholine | LPC |
|  | Phosphatidylcholine | PC |
|  | Lysophosphatidylethanolamine | LPE |
|  | Phosphatidylethanolamine | PE |
|  | Lysophosphatidylglycerol | LPG |
|  | Phosphatidylglycerol | PG |
|  | Cardiolipin | CL |
|  | Phosphatidylinositol | PI |
|  | Lysophosphatidylserine | LPS |
|  | Phosphatidylserine | PS |
| Sphingolipids | Ceramide | Cer |
|  | Neutral glycosphingolipid | HexCer |
|  | Sphingomyelin | SM |
|  | Sulfonolipid | SL |
|  | Sphinganine | SPB |
| Sterol Lipids | Cholesteryl ester | CE |
|  | Sitosterol ester | SE |
|  | Cholic acid | Bile Acid/ST |
|  | Acylhexosyl sitosterol | ASG |
